# *Arabidopsis thaliana WRKY25* Transcription Factor Mediates Oxidative Stress Tolerance and Regulates Senescence in a Redox-Dependent Manner

**DOI:** 10.3389/fpls.2019.01734

**Published:** 2020-01-23

**Authors:** Jasmin Doll, Maren Muth, Lena Riester, Sabrina Nebel, Justine Bresson, Hsin-Chieh Lee, Ulrike Zentgraf

**Affiliations:** Center for Plant Molecular Biology (ZMBP), University of Tuebingen, Tuebingen, Germany

**Keywords:** Arabidopsis, transcription factor network, WRKY factors, oxidative stress tolerance, redox-dependent DNA-binding, leaf senescence

## Abstract

Senescence is the last developmental step in plant life and is accompanied by a massive change in gene expression implying a strong participation of transcriptional regulators. In the past decade, the WRKY53 transcription factor was disclosed to be a central node of a complex regulatory network of leaf senescence and to underlie a tight multi-layer control of expression, activity and protein stability. Here, we identify WRKY25 as a redox-sensitive up-stream regulatory factor of *WRKY53* expression. Under non-oxidizing conditions, WRKY25 binds to a specific W-box in the *WRKY53* promoter and acts as a positive regulator of *WRKY53* expression in a transient expression system using Arabidopsis protoplasts, whereas oxidizing conditions dampened the action of WRKY25. However, overexpression of *WRKY25* did not accelerate senescence but increased lifespan of Arabidopsis plants, whereas the knock-out of the gene resulted in the opposite phenotype, indicating a more complex regulatory function of WRKY25 within the WRKY subnetwork of senescence regulation. In addition, overexpression of WRKY25 mediated higher tolerance to oxidative stress and the intracellular H_2_O_2_ level is lower in *WRKY25* overexpressing plants and higher in *wrky25* mutants compared to wildtype plants suggesting that WRKY25 is also involved in controlling intracellular redox conditions. Consistently, *WRKY25* overexpressers had higher and *wrky* mutants lower H_2_O_2_ scavenging capacity. Like already shown for WRKY53, MEKK1 positively influenced the activation potential of WRKY25 on the *WRKY53* promoter. Taken together, WRKY53, WRKY25, MEKK1 and H_2_O_2_ interplay with each other in a complex network. As H_2_O_2_ signaling molecule participates in many stress responses, WRKK25 acts most likely as integrators of environmental signals into senescence regulation.

## Introduction

Senescence is the last step during plant development and is genetically programmed to maximize the remobilization of nutrients out of the senescing tissue into developing parts of the plants before organs finally die. Before anthesis, sequential leaf senescence leads to the reallocation of mineral, nitrogen and carbon sources from older leaves to newly developing non-reproductive organs. After anthesis, monocarpic leaf senescence is launched and governs the nutrient repartitioning to the now developing reproductive organs and, therefore, has a critical impact on yield quality and quantity. Induction and progression of leaf senescence is mainly achieved by switching-on genes involved in degradation and mobilization of macromolecules and turning-off genes related to photosynthesis. A temporal transcript profiling, using microarrays with high-resolution covering 22 time points of a defined leaf of *Arabidopsis thaliana* during onset and progression of leaf senescence, revealed a distinct chronology of events (Breeze et al., 2011). Remarkably, the first processes to be activated are autophagy and transport followed by reactions to reactive oxygen species (ROS) and subsequently to abscisic acid (ABA) and jasmonic acid (JA). This clearly indicates that ROS, ABA and JA are important early signals in leaf senescence. In consistence, intracellular hydrogen peroxide contents increase during bolting and flowering of Arabidopsis plants when monocarpic senescence is induced (Zimmermann et al., 2006) while decreasing hydrogen peroxide levels lead to a delay of the onset of leaf senescence (Bieker et al., 2012).

These massive changes in the transcriptome suggest a central role for transcriptional regulators. The two transcription factor families of WRKY and NAM-, ATAF-, and CUC-like (NAC) factors, which largely expanded in the plant kingdom, are overrepresented in the senescence transcriptome of Arabidopsis (Guo et al., 2004) and appear to be ideal candidates for regulatory functions. Several members of both families play important roles in senescence, not only in Arabidopsis but also in other plant species (Miao et al., 2004; Uauy et al., 2006; Ülker et al., 2007; Kim et al., 2009; Breeze et al., 2011; Yang et al., 2011; Besseau et al., 2012; Wu et al., 2012; Gregersen et al., 2013).

The WRKY transcription factor family of *A. thaliana* consists of 75 members, subdivided into three different groups according to their protein motifs and domains (Eulgem et al., 2000; Rushton et al., 2010). Many WKRY factors are activated after pathogen attack but also in response to abiotic stress (for review see Birkenbihl et al., 2017; Jiang et al., 2017). Moreover, members of all three groups are involved in senescence regulation and many of these react to ROS, SA and JA signals indicating a cross-talk between stress responses and senescence. Besides this cross-talk to stress responses, the *WRKY53* upstream regulator REVOLUTA mediates a redox-related communication between early leaf patterning and senescence as REVOLUTA is involved in both processes (Xie et al., 2014; Kim et al., 2017).

Interestingly, almost all members of the WRKY family contain one or more W-boxes (the consensus binding motif TTGACC/T of all WRKY factors) in their promoters, pointing to a WRKY transcriptional network (Dong et al., 2003; Llorca et al., 2014). Even though all WRKYs bind to these consensus sequences, there appears to be a selectivity of specific factors for specific boxes most likely due to the surrounding sequences (Rushton et al., 2010; Brand et al., 2013; Potschin et al., 2014). However, besides regulating transcription of each other, WRKY factors are also able to form heterodimers, leading to a change in DNA-binding specificity (Xu et al., 2006). In addition, many other proteins interact physically with WRKY proteins influencing their activity and stability (for review see Chi et al., 2013). One central node in the WRKY network regulating early senescence is WRKY53. WRKY53 underlies a tight regulation governed by multi-layer mechanisms to control expression, activity and protein stability. When leaf senescence is induced, the *WRKY53* gene locus is activated by the histone modifications H3K4me2 and H3K4me3 (Ay et al., 2009; Brusslan et al., 2012), whereas DNA methylation remains unchanged and overall very low (Zentgraf et al., 2010). At least 12, most likely even more, proteins are able to bind to the promoter of *WRKY53* (GATA4, AD-Protein, WRKY53 itself, several other WRKYs, MEKK1, REVOLUTA, WHIRLY1) and influence the expression of *WRKY53* (Miao et al., 2004; Miao et al., 2007; Miao et al., 2008; Potschin et al., 2014; Xie et al., 2014; Ren et al., 2017, unpublished results). All these factors are involved in senescence regulation but it is still unclear whether they all bind at exactly the same time, whether they compete with each other, or whether they form higher order complexes. Except for the WRKYs that bind to the W-boxes in the *WRKY53* promoter, all other proteins have different binding motifs so that in principle a simultaneous interaction would be possible. The *WRKY53* promoter contains at least three W-boxes, which show preferential binding activities for different WRKY factors but competition would be also a mean of regulation. Moreover, WRKYs can also form heterodimers, which makes the situation even more complicated. However, all these aspects need further investigations. For MEKK1, it has already been shown that it can interact with WRKY53 and AD-Protein on the protein level (Miao et al., 2007; Miao et al., 2008). Whether WRKY53 or other WRKYs compete with AD-protein for MEKK1 interaction or whether they form higher order complexes is currently analyzed in more detail. These findings have been compiled in a model (Zentgraf et al., 2010) und smaller subnetworks have already schematically drawn for some candidates like WRKY18, REVOLUTA or WHIRLY1 (Potschin et al., 2014; Xie et al., 2014; Ren et al., 2017).

Moreover, the WRKY53 protein also directly interacts with a histone deacetylase 9 (HDA9) to recruit POWERDRESS and HDA9 to W-box containing promoter regions to remove H3 acetylation marks and thereby suppress the expression of key negative senescence regulators (Chen et al., 2016). This clearly suggests that WRKY53 itself is also involved in changing epigenetic marks of senescence regulators in a feedback loop. Phosphorylation by the MAP kinase kinase kinase MEKK1 or the interaction with the epithiospecifier ESP/ESR directly influences the DNA-binding activity of WRKY53 (Miao et al., 2007; Miao and Zentgraf, 2007). On top of that, the E3 ubiquitin ligase UPL5 tightly controls the protein amount of WRKY53 (Miao and Zentgraf, 2010). The complexity of the WRKY network is illustrated by the fact that one and the same WRKY factor, namely WRKY18, acts as upstream regulator, downstream target and protein interaction partner of WRKY53 (Potschin et al., 2014).

In order to unravel the molecular mechanisms of the senescence-regulating WRKY network in more detail, we screened the W-boxes of the *WRKY53* promoter for DNA-protein interactions with other leaf senescence-associated WRKY proteins and tested their impact on *WRKY53* expression using a transient expression system in Arabidopsis protoplasts. Here we used WRKYs which are expressed in leaves during onset and progression of senescence and which belong to the three different subgroups of the WRKY family, namely WRKY18 (group IIa), WRKY25 (group I) and WRKY53 itself (group III). Out of the 15 WKRYs analyzed by an ELISA-based DNA-protein interaction assay and reporter gene expression assays, WRKY18 had a very strong binding affinity to all W-boxes of the *WRKY53* promoter but a very low selectivity. Moreover, WRKY18 was characterized to be the strongest negative regulator of *WRKY53* expression (Potschin et al., 2014). Besides WRKY18, WRKY25 was one of the strongest interaction partners of the *WRKY53* promoter but in this case turned out to be a strong positive regulator of *WRKY53* expression. Therefore, we wanted to analyze the interplay between WRKY53 and WRKY25 in more detail. Here we could show that DNA-binding as well as transcriptional activation potential of WRKY25 is dependent on the redox conditions. Intracellular hydrogen peroxide concentrations are altered in plants with altered *WRKY25* expression and the *WRKY25* overexpressing plants are more tolerant against oxidative stress. WRKY25 appears to foster the activation of the H_2_O_2_-mediated expression of the transcription factors *WRKY18* but dampens the H_2_O_2_-response of *WRKY53*, *ZAT12*, and *ANAC092* in mature leaves. However, contradicting its positive effect on *WRKY53* expression and the senescence phenotype of the *WRKY53* overexpressing plants, *WRKY25* overexpressing plants exhibited a delayed senescence phenotype, whereas *wrky25* mutant plants showed slightly accelerated senescence. This clearly points to a more complex regulatory network. Moreover, the influence of MEKK1 as modulator of WRKY53 activity on the action of WRKY25 was tested.

## Results

### WRKY25 Binds Directly to the Promoter of *WRKY53 in* a Redox-Sensitive Manner

**Figure 1:**
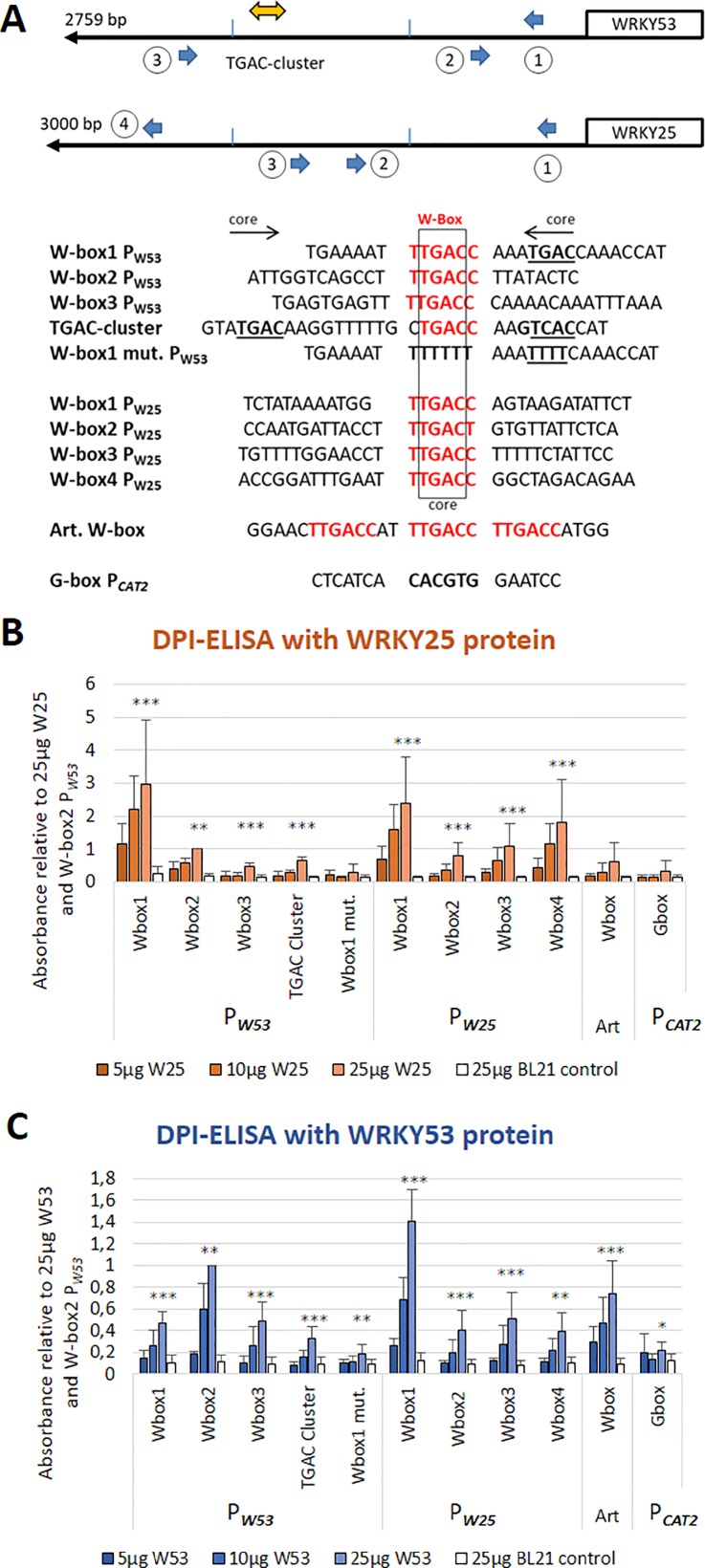
WRKY25 is able to bind directly to the *WRKY53* promoter. **(A)** Schematic drawing of positions and sequences of the W-boxes in the *WRKY53* and *WRKY25* promoters used for DPI-ELISAs; perfect W-box motifs are highlighted in red; the TGAC core sequence is indicated in bold and underlined; the direction of the motif is indicated by the arrows. An artificial sequence containing three perfect W-boxes was used as a positive control (Art. W-box), a G-box of the *CATALASE2* promoter was used as a negative control. **(B)** DPI-ELISA with different amounts of crude extracts of *E. coli* BL21 cells expressing WRKY25 proteins and different biotinylated DNA fragments. **(C)** DPI-ELISA with different amounts of crude extracts of *E. coli* BL21 cells expressing WRKY53 proteins and different biotinylated DNA fragments. Absorbance values are indicated relative to values of 25 μg WRKY25 or WRKY53 to W-box2 P*_W53_*, respectively, (mean values ± SD, n = 3-4). Kruskal–Wallis-test was performed for statistically significant differences of all values compared to 25 μg BL21 control (*P ≤ 0.05, **P ≤ 0.01, ***P ≤ 0.001).

Out of the 15 WKRYs analyzed by an ELISA-based DNA-protein interaction assay, WRKY18 (group IIa) and WRKY25 (group I) had a very strong binding affinity. In contrast to WRKY18, which strongly binds to all W-boxes in the *WRKY53* promoter (Potschin et al., 2014), WRKY25 also had a strong binding activity but selectively bound to W-box1, to a much lesser extend to W-box2 and 3, the TGAC cluster and an artificial 3× W-box (**Figures 1A, B**). Binding was completely abolished when W-box1 was mutated or an unrelated G-box motive was coupled to the ELISA plates. All binding reactions increased with protein concentrations and no binding could be detected with crude extracts of *E. coli* cells expressing no recombinant protein. Both proteins were present approximately to the same extent in *E. coli* crude extracts (**Figure S1**). As many WRKY factors signal back to their own promoters in positive or negative feedback loops, we also tested whether WRKY25 can bind to the W-boxes in its own promoter. Here, WRKY25 was able to bind to W-box1 and 4, whereas W-box2 and 3 exhibited lower binding affinities. *Vice versa*, WRKY53 also bound preferentially to W-box 1 of the *WRKY25* promoter but to W-box2 of its own promoter, as already shown before (**Figure 1C**, Potschin et al., 2014). This indicates that according to DNA-binding, there is a cross-regulation between both genes and both genes are regulated by feedback mechanisms.

**Figure 2:**
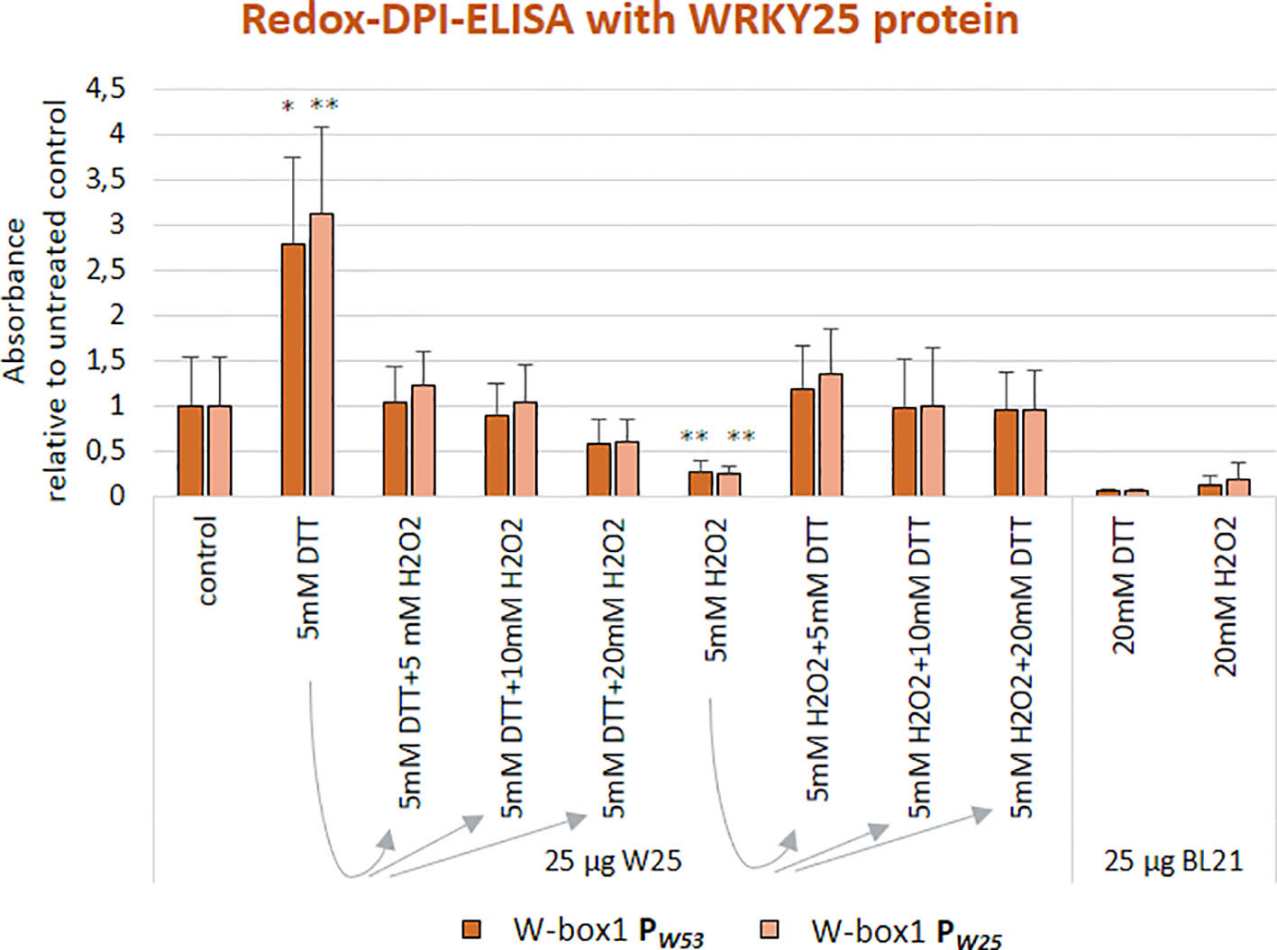
WRKY25 binding to the *WRKY53* promoter is redox-dependent. Redox-DPI-ELISA with 25 μg of crude extracts of *E. coli* BL21 cells expressing WRKY25 proteins and the 5'biotinylated annealed oligonucleotides W-box1 P*_W53_* and W-box1 P*_W25_*. Protein extracts were reduced or oxidized by addition of either DTT or H_2_O_2_ to examine a redox-dependent binding of WRKY25. A fraction of the DTT-reduced proteins was re-oxidized by addition of increasing H_2_O_2_ concentrations to prove the reversibility of the redox effect. The same procedure was applied to the H_2_O_2_-oxidized proteins using increasing amounts of DTT. Absorbance values are indicated relative to control without treatment (mean values ± SD, n = 4). Kruskal–Wallis-test was performed for statistically significant differences of all values compared to control (*P ≤ 0.05, **P ≤ 0.01).

We already know for a long time that *WRKY53* expression can be induced by H_2_O_2_ treatment (Miao et al., 2004; Xie et al., 2014). As the WRKY25 protein contains two potentially redox-sensitive zinc-finger DNA-binding domains, it is an excellent candidate for direct redox regulation (Arrigo, 1999). Therefore, we wanted to test whether the WRKY25 DNA-binding reaction is sensitive to reducing or oxidizing agents and analyzed the ability of WRKY25 to bind to W-box1 of the *WRKY53* promoter and W-box1 of the *WRKY25* promoter under different redox conditions. Whereas reducing conditions (DTT addition) clearly and significantly increased DNA-binding ability to both W-boxes, oxidizing conditions (H_2_O_2_ addition) significantly reduced the binding activity in comparison to standard binding conditions (**Figure 2**). In order to test whether this redox-dependent binding can be driven back and forth when redox conditions change, we added increasing amounts of H_2_O_2_ to the DTT pre-treated binding reactions and *vice versa*. Both redox-related changes in DNA-binding activity of WRKY25 were reversible indicating that WRKY25 can directly adapt its DNA-binding activity to the redox status of the cell. However, not all WRKYs show this redox-sensitivity, e.g. WRKY18 appears to be insensitive, whereas WRKY53 DNA-binding seems to be diminished under oxidizing and reducing conditions, but this reduction was not statistically significant (**Figure S2**).

### WRKY25 Acts as Positive Regulator of *WRKY53* Expression Under Non-Oxidizing Conditions

**Figure 3:**
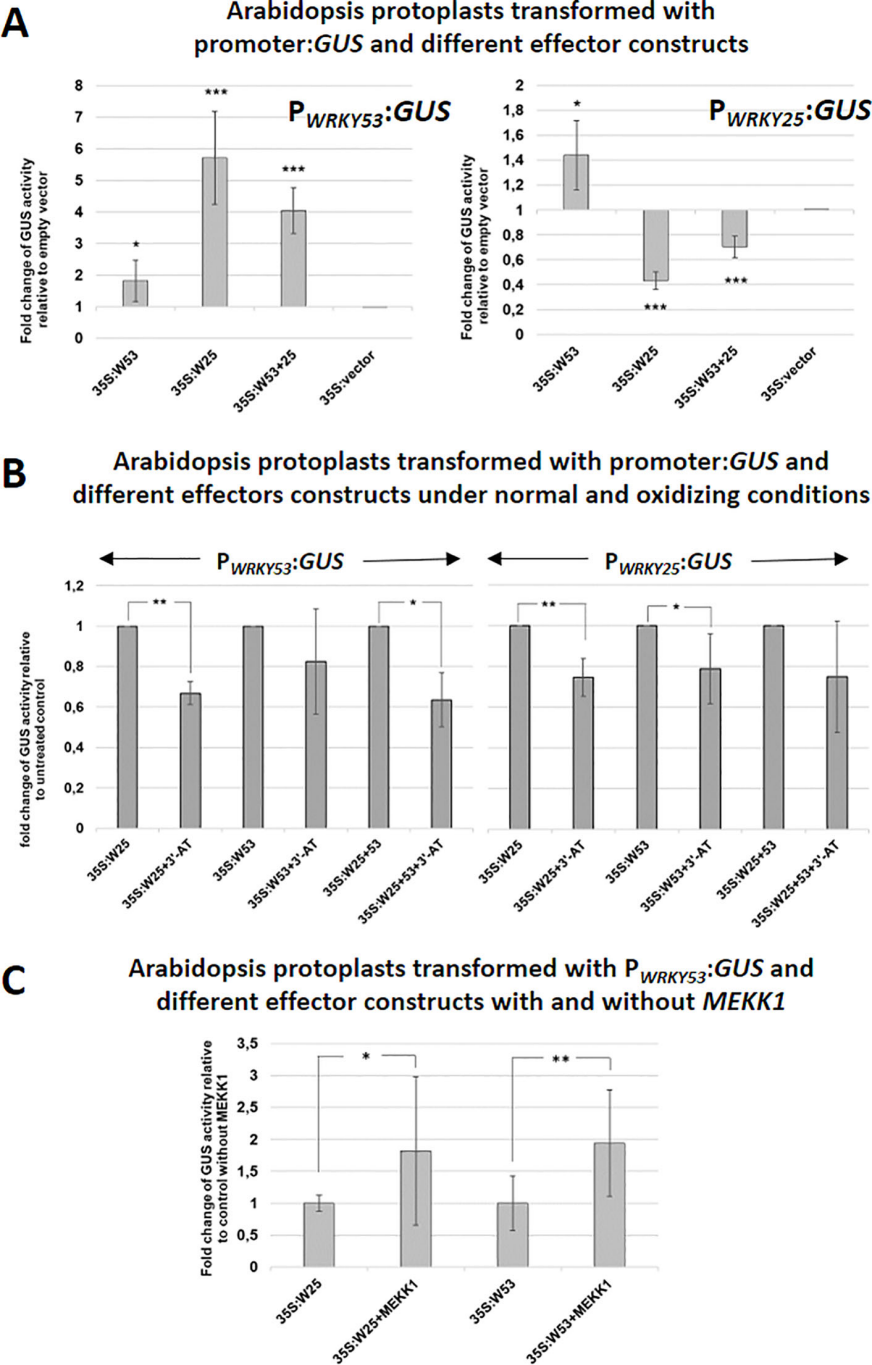
WRKY25 positively regulates *WRKY53* under non-oxidizing conditions. **(A)** Arabidopsis protoplasts were transiently transformed with 5 µg of effector-, 5 µg of reporter-plasmid DNA and 0.1 µg of a luciferase construct for normalization. A 2.8-kbp-fragment of the *WRKY53* promoter and a 3.0-kbp-sequence of the *WRKY25* promoter fused to the *GUS* gene were used as reporter constructs. 35S:*WRKY25* and 35S:*WRKY53* constructs were used as effector plasmids. GUS activity was measured on the next day. The values are presented relative to the empty vector control (mean values ± SD, n = 6 independent transfromations). One sample t-test was performed, (*P ≤ 0.05, **P ≤ 0.01, ***P ≤ 0.001) **(B)** GUS assays were performed with protoplasts, which were simultaneously incubated overnight with 10 mM 3'-AT to inhibit catalase activities leading to higher H_2_O_2_ level. The values are presented relative to the untreated control transformations (mean values ± SD, n = 3–7 independent transformations). One sample t-test was performed, (*P ≤ 0.05, **P ≤ 0.01). **(C)** Co-transformation assays with 35S:*MEKK1* were performed. The values are presented relative to transformation without MEKK1 (mean values ± SD, n = 4–5 independent transformations). Kruskal–Wallis-test was performed, (*P ≤ 0.05, **P ≤ 0.01).

To investigate, how WRKY25 affects the expression of *WRKY53* and *vice versa*, we performed a transient co-transformation of *WRKY53* or *WRKY25* promoter:*GUS* constructs with 35S:*WRKY25* and 35S:*WRKY53* effector constructs, respectively, using an Arabidopsis protoplast system (**Figure 3A**). The protoplast system was used to confirm the identified DNA-Protein interactions of the *in vitro* assay also *in vivo*. However, it is clear that the protoplast system is still an artificial system not taking into account development cues, but it can provide inside into the possible basic regulatory mechanisms. Using this *in vivo* system, the WRKY25 effector significantly up-regulated promoter *WRKY53*-driven *GUS* expression. In contrast, it down-regulated *GUS* expression driven by its own promoter pointing to a negative feedback regulation. The WRKY53 effector slightly activated reporter gene expression driven by its own promoter as already described before (Potschin et al., 2014). Surprisingly, WRKY53 had only low effects (1.4-fold) on reporter gene expression driven by the promoter of *WRKY25* (**Figure 3A**), even though WRKY53 is able to bind strongly to the W-boxes of this promoter (**Figure 1C**) indicating that strong binding does not necessarily mean that gene expression is highly affected. If both effector constructs were co-expressed, additive effects were detected leaving the question open whether or not heterodimers are formed.

As DNA-binding of WRKY25 was redox-sensitive, we wanted to find out, whether also target gene expression is affected by the redox conditions. Since we wanted to change the redox conditions within a physiological range, we did not treat protoplasts directly with high amounts of H_2_O_2_. Instead, we developed a transient expression system using Arabidopsis protoplast in the presence of 3-Amino-Triazol (3´-AT), which inhibits catalase function, and would therefore provoke physiological changes in intracellular H_2_O_2_ levels. Inhibition of catalase activity was almost complete and leads to increasing concentrations of H_2_O_2_ in the cells, but had no effect on the GUS activity measurement (**Figure S3**). Using this assay, WKRY25 effector proteins were significantly less efficient under oxidizing conditions, most likely due to lower DNA-binding affinity. WRKY53 effector proteins appeared also to be less efficient, but the effect was only significant for the WRKY25 promoter, not for its own. The effects were still significant when a combination of both effectors constructs was used (**Figure 3B**).

### MEKK1 Increases the Effect of WRKY25 Proteins on Promoter of *WRKY53* Driven Gene Expression

As expression of *WRKY53* is enhanced by a direct binding of MEKK1 to the promoter region of *WRKY53* and a protein–protein interaction between WRKY53 and MEKK1 leads to phosphorylation of WRKY53 (Miao et al., 2007), we tested whether WRKY25 activity can also be enhanced by adding a 35S:*MEKK1* construct as additional effector in a protoplast co-transformation assay. Indeed, the presence of the MEKK1 protein significantly increased *WRKY53* promoter-driven reporter gene expression by WRKY25 to approximately the same extent as MEKK1 presence exhibits on WRKY53 activity itself (**Figure 3C**). Thus, MEKK1 interplay with WRKY factors is not restricted to WRKY53, but appears to be a more general phenomenon. First evidence for a direct protein-protein interaction between several WRKY factors and MEKK1 was obtained in a Yeast-Split-Ubiquitin system, in which many, but not all tested WRKYs could interact with MEKK1 (data not shown). WRKY18, which acted as a repressor on promoter *WRKY53*-driven reporter gene expression (Potschin et al., 2014), even changed its activity in the presence of MEKK1 from a repressor to an activator (**Figure S4A**). Moreover, we tested the role of MEKK1 in senescence regulation. As *MEKK1* knock-out plants die before they develop the first true leaves, we used an estradiol-inducible amiRNA*_MEKK1_* line to knock-down MEKK1 by treatment with 3 µM ß-estradiol or mock every 7 days starting on day 25 after germination. In this system, knock-down of MEKK1 can be controlled by GFP expression, which is under the control of the same amiRNA (Li et al., 2013). Here we could show that conditional knock-down of *MEKK1* in plants exhibit an accelerated senescence phenotype (**Figures S4B–D**). Taken together, MEKK1 appears to act as negative regulator of senescence at least in part by modulating the activity of different WRKY factors. However, whether the interaction with WRKY25 or WRKY18 is direct as it is for WRKY53, or is mediated by the classical MAPK pathway, still has to be elucidated.

### WRKY25 Is Involved in Senescence Regulation

**Figure 4:**
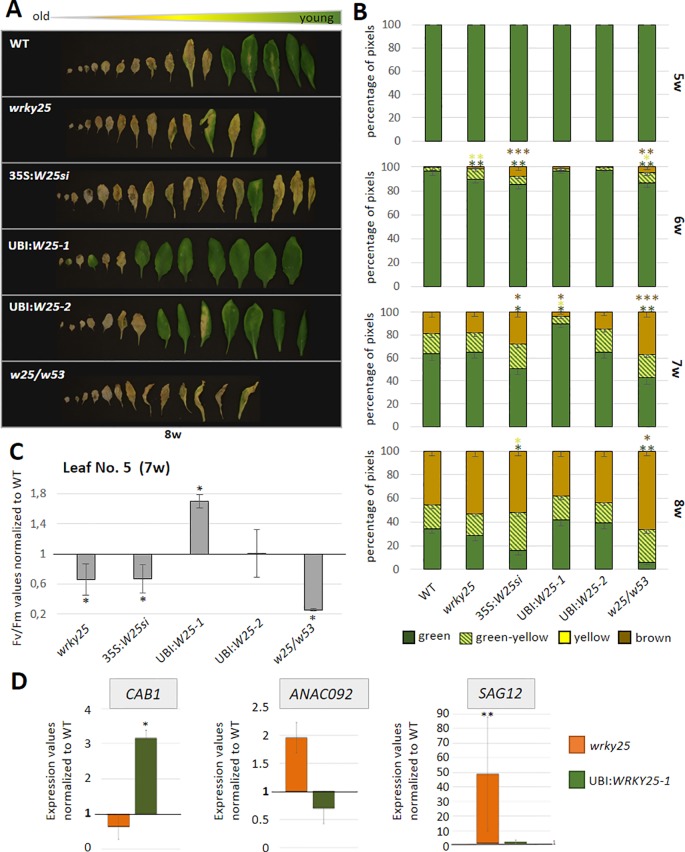
Senescence phenotypes of *WRKY25* transgenic and mutant lines. **(A)** Col-0 wildtype (WT), *wrky25* mutant (*wrky25*), *WRKY25* overexpressing (UBI:*W25-1* and UBI:*W25-2*), *WRKY25* silenced (35S:*W25si*) and *wrky25-wrky53* double-knock-out (*w25/w53*) plants were analyzed over development. A photograph of rosette leaves of 8-week-old plants sorted according to their age is shown. **(B)** Quantitative evaluation of leaf senescence by categorizing individual leaves of at least six plants into four groups according to their color: green, green leaves starting to get yellow (green-yellow), completely yellow leaves (yellow) and dead and/or brown leaves (brown/dry). The percentage of each group with respect to total leaf numbers are presented (mean values ± SE, n = 6). **(C)** Fv/Fm values were measured with PAM for leaves of position 5 of 7-week-old plants (mean values ± SE, n = 6). One sample t-test was performed for statistical differences of all values compared to Col-0 (*P ≤ 0.05) **(D)** Expression of the senescence associated marker genes *ANAC092*, *CAB1, SAG12* were analyzed by qRT-PCR and normalized to the expression of the *ACTIN2* gene. *SAG12* in 6-week and *ANAC092* and *CAB1* in 7-week-old plants. Shown are *wrky25* and UBI:*W25-1* plants normalized to Col-0 (mean values ± SE, n = 3). Kruskal–Wallis-test was performed for statistically significant differences (*P ≤ 0.05, **P ≤ 0.01, ***P ≤ 0.001).

**Figure 5:**
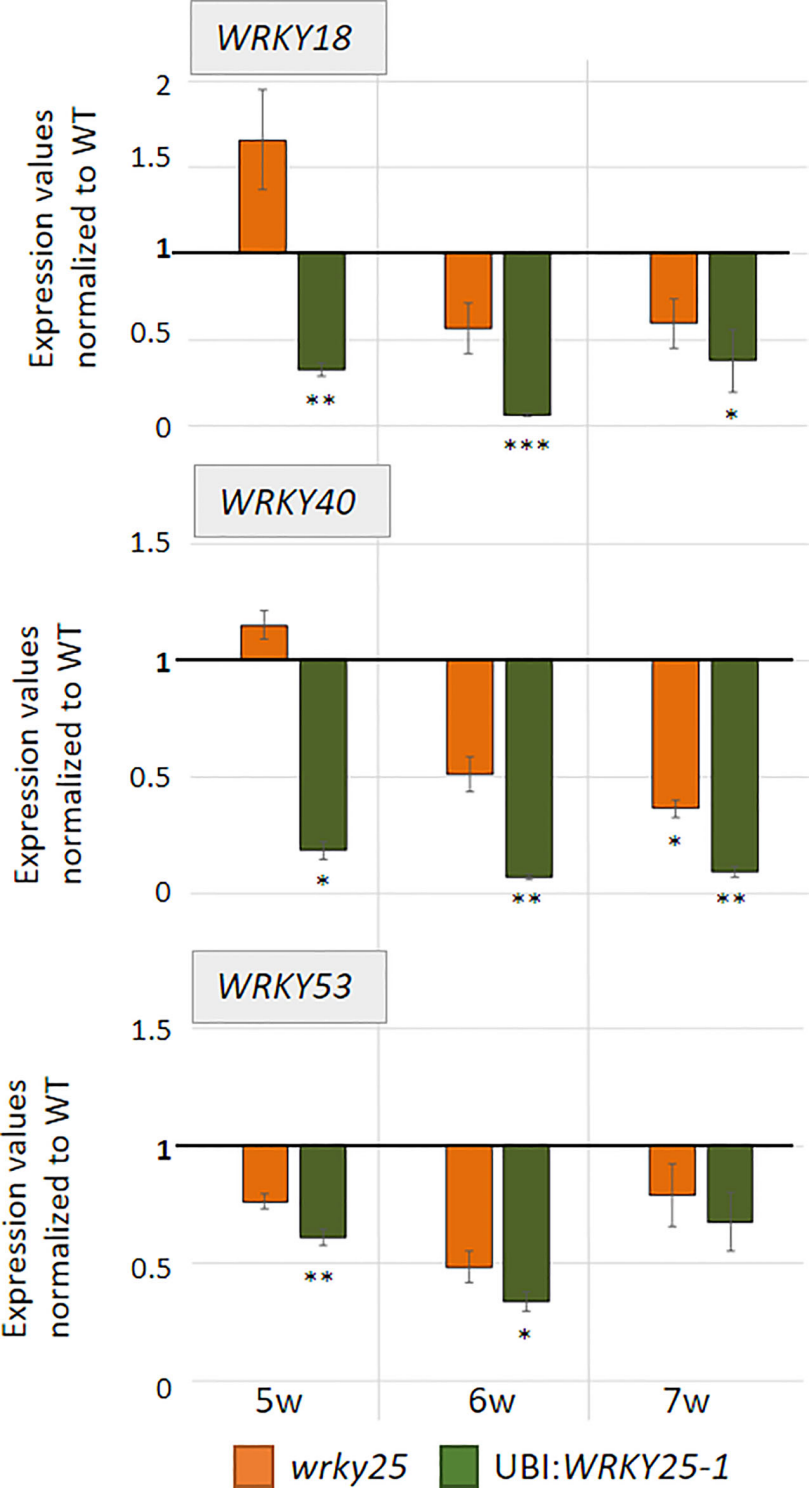
WRKY genes expression analyses. Expression of different WRKY genes (*WRKY53*, *WRKY18*, *WRKY40*) were analyzed in Col-0 (WT), *wrky25* mutant and *WRKY25* overexpression line (UBI : *WRKY25-1)* by qRT-PCR and normalized to the expression of the *ACTIN2* gene. Three pools were analyzed; one pool consists of leaf No. 6 and 7 of two different plants. In week 7, only two pools of the *35S:W25si* plant line and of the *wrky25* line were analyzed but here with six technical replicates. Expression values were normalized to Col-0 and Col-0 was set to 1 (mean values, n = 3, ± SE). Kruskal–Wallis-test was performed for statistically significant differences of all value compared to Col-0 (*P ≤ 0.05, **P ≤ 0.01, ***P ≤ 0.001).

To evaluate the participation of WRKY25 in senescence regulation, we analyzed plants with a T-DNA insertion in the *WRKY25* gene lacking a functional WRKY25 protein and *WRKY25* overexpressing plants. A T-DNA insertion in the last of five exons of *WRKY25* (SAIL_529_B11) was confirmed by PCR and expression of *WRKY25* was analyzed by qRT-PCR (**Figure S5B**). Moreover, for overexpression of *WRKY25,* we first transformed plants using a 35S:*WRKY25* construct. However, qRT-PCR revealed that *WRKY25* was not overexpressed; in contrast, the endogenous gene expression was severely silenced throughout plant development (**Figure S5A**) so that we used this line as knock-down line (35S*:WRKY25si*) to confirm the results of the *wrky25* mutant plants. In a second attempt, we used the *UBIQITIN10* promoter for more moderate overexpression and we created two independent plant lines overexpressing *WRKY25* to different extents with different transgene expression levels (**Figure S5B**). In addition, double-knock-out mutants were created by crossing the single mutant lines *wrky25* (SAIL_529_B11) and *wrky53* (SALK_034157; Miao et al., 2004) with each other. F2 progenies were screened for homozygous double-knock-out plants. In order to compare leaves of the same position within the rosette for senescence symptoms, leaves were color-coded during development (Bresson et al., 2018). Altered *WRKY25* expression had almost no effect on the speed of the general development of the plants (**Figure S6**). Bolts appeared at approximately week 5 in all lines, first flowers at approximately week 6 and first siliques also developed synchronously. However, leaf size slightly increased in the overexpression lines whereas leaves of the *wrky25* mutant, the *wrky25/wrky53* double-knock-out plants and the *WRKY25* silenced line were slightly smaller. To evaluate senescence in detail, we sorted the rosette leaves of all lines by the color code according to their age to compare the respective leaves with each other. A typical example of rosette leaves of 8-week-old plants is shown in **Figure 4A**. However, as there are always differences between individual plants of one line, a statistical analysis of at least six plants was done by grouping the leaves into four categories by an automated colorimetric assay (ACA; Bresson et al., 2018) according to their leaf color (green; green/yellow; fully yellow and brown/dry) from weeks 5 to 8 (**Figure 4B**). The photosynthetic status of the plants was analyzed using a Pulse-Amplitude-Modulation (PAM) method (**Figure 4C**). Amongst the chlorophyll fluorescence imaging parameters, the Fv/Fm ratio is reflecting the maximal quantum yield of PSII photochemistry. Moreover, the expression of the senescence marker genes *CHLOROPHYLL A/B BINDING PROTEIN 1* (*CAB1*) being downregulated, the NAC transcription factor *ANAC092*, and *SENESCENCE-ASSOCIATED GENE 12* (*SAG12)* being upregulated were analyzed by qRT-PCR (**Figure 4D**). In comparison to Col-0 wildtype plants, *WRKY25* overexpressing plants showed significantly delayed visible senescence symptoms, which was in consistence with a delay in the decrease of the Fv/Fm ratio measured in leaf No. 5 and leaf No. 10 (**Figure 4C**, **Figure S7**). Furthermore, a higher expression of *CAB1* in 7-week-old plants as well as a lower expression of *ANAC092* and *SAG12* in 6-week-old plants of *WRKY25* overexpressing line compared to wildtype confirmed a delayed senescence phenotype. In contrast, senescence and loss of photosynthetic activity was accelerated in the *wrky25* mutant plants and the 35S*:WRKY25si* line (**Figures 4A–C**). Higher up-regulation of *ANAC092* and *SAG12* expression in 6-week-old plants and lower *CAB1* expression in 7-week-old plants in *wrky25* mutant line clearly indicate an accelerated senescence phenotype (**Figure 4D**). Remarkably, the expression of *WRKY53* was lower in the *WRKY25* overexpressing as well as in the *wrky25* mutant lines in comparison to WT (**Figure 5**), suggesting a more complex regulation of WRKY53 expression during development. Moreover, the expression of two tested WRKY genes (*WRKY18* and *WRKY40*) was antagonistic in *WRKY25* overexpressing and mutant plants, but only at week 5; at later stages also these two WRKY genes were down-regulated in both lines. We have chosen WRKY40 as it is also expressed in senescent leaf tissue. WRKY40 was also shown to regulate *WRKY53* expression in a negative way, but to a lesser extent. Moreover, it is the closest relative of WRKY18 also belonging to group IIa so that we can see whether regulatory processes are group specific which appears to be the case. This clearly indicates that no simple regulatory circuits are in place between these WRKY proteins and genes. WRKY25 as well as WRKY53 and WRKY18 appear to be part of a WRKY subnetwork, which is embedded in the overall complex senescence regulatory network. Interfering on the expression of one WRKY gene can lead to an imbalance in the subnetwork, which might explain that mutant and overexpressing plants showed the same effects on the expression of specific WRKYs. Taken together, WRKY25 appears to be part of the WRKY subnetwork and a redox-sensitive negative regulator of senescence.

### WRKY25 Mediates Tolerance Against Oxidative Stress

**Figure 6:**
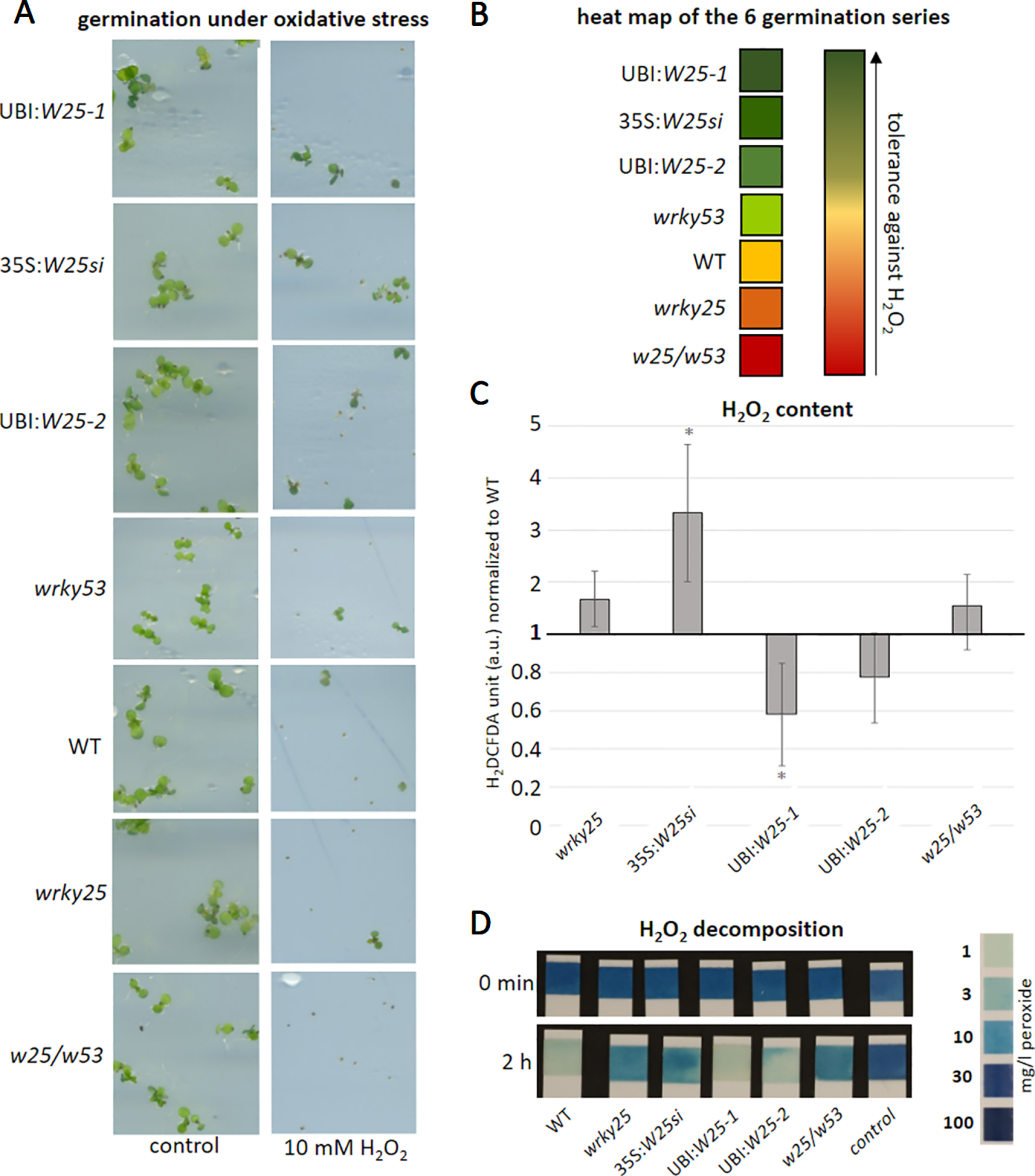
WRKY25 mediates H_2_O_2_ tolerance. **(A–B)** Col-0 (WT), *wrky25* mutant, *wrky53* mutant, *WRKY25* overexpressing (*UBI:W25-1; UBI:W25-2*) and *WRKY25* silenced (*35S:W25si*) as well as the double-knock-out plants (*w25/w53*) were sown on ½ MS plates with and without 10 mM H_2_O_2_. A minimum of 30 seeds were put onto the plates and the experiment repeated 6 times (n = 6) **(A)** shows a representative sector of the plates with and without H_2_O_2_ in media. **(B)** summarizes the six independent experiments in a heat map. Dark green means the most tolerant against H_2_O_2_, dark red the most sensitive towards H_2_O_2_. **(C)** H_2_O_2_ content was measured over plant development using H_2_DCFDA fluorescence; leaves No. 8 of 8-week-old plants are shown. Fluorescence is indicated in arbitrary units (a.u.), normalized to leaf weight and expressed relative to WT ( ± SE, n = 4). One Sample t-test was performed for statistical differences of all values compared to WT (*P ≤ 0.1) **(D)** Leaf discs of leaf No. 5 of 6-week-old plants were incubated in a 30 mg/l H_2_O_2_ solution. As control H_2_O_2_ solution without leaf discs was measured. At timepoint 0 min and 2 h the decomposition of H_2_O_2_ was determined using commercially available peroxide sticks, color scale for H_2_O_2_ content is provided on the right.

As WRKY25 action appears to be redox-sensitive, we wanted to analyze whether WRKY25 is also involved in the response to oxidative stress or plays a role in the signaling of H_2_O_2_ *in planta*. Therefore, we germinated seeds of WT, *wrky25* mutant, *wrky53* mutant, 35S:*WRKY25si*, the *WRKY25* overexpressing lines as well as the double mutant *wrky25/wrky53* on plates containing 10 mM H_2_O_2_ (**Figure 6A**). After 7 to 10 days, the percentages of green seedlings per total seedling numbers were counted. The experiment was repeated six times and the outcome of these series were summarized in a heat map showing the tolerance against H_2_O_2_ (**Figure 6B**). The germination rate on the control plates without H_2_O_2_ was almost 100% for all plant lines used. The 35S:*WRKY25si* and the *WRKY25* overexpressing lines *(*UBI:*W25-1* and UBI:*W25-2*) germinated much better on H_2_O_2_ plates in comparison to WT as well as the *wrky53* single mutant. In contrast, the *wrky25* as well as the *wrky25/wrky53* mutant seeds germinated significantly worse compared to WT. Therefore, WRKY25 seems to mediate a higher tolerance against H_2_O_2_. Even though gene silencing was clearly shown from the late seedling stage until the end of leaf development (**Figure S5**), the *wrky25* mutant and the 35S:*WRKY25si* knock-down line behave different in this experiment. This behavior can only be explained by the assumption that during very early stages of germination, the 35S:*WRKY25* line overexpressed the transgene but gene silencing was not yet established at this very early time points. This could indeed be confirmed by expression analyses of *WRKY25* in the 35S:*WRKY25si* line using qRT-PCR in very early germination states (4 and 7 day old seedlings, **Figure S5A**).

In order to test whether this tolerance is due to higher antioxidative capacities in these lines, we measured intracellular H_2_O_2_ contents of leaf No. 8 in 8-week-old plants of these lines (**Figure 6C**). Less intracellular H_2_O_2_ was measured in the overexpressing lines, while more H_2_O_2_ appears to be present in the mutants and the silenced line in comparison to WT (**Figure 6C**). Moreover, the H_2_O_2_ scavenging capacity of leaf discs of the different lines was tested by incubating these discs for 2 h in H_2_O_2_ solution and measure the remaining H_2_O_2_ using peroxide strips (**Figure 6D**). As expected, the antioxidative capacity of the *WRKY25* overexpressing lines was slightly higher, whereas scavenging in the mutant and silencing lines was lower. Taken together, WRKY25 does not only mediate a higher tolerance against oxidative stress but is also involved in the regulation of intracellular H_2_O_2_ levels, at least in later developmental stages. This might also contribute to the negative effect of WRKY25 on senescence since H_2_O_2_ acts as signaling molecule to induce senescence and, most likely, also participates in membrane deterioration and lipid peroxidation processes in later stages (Chia et al., 1981). The conclusions on the role of WRKY25 in senescence-related redox signal transduction is further supported by a dark-induced senescence experiment including *wrky25*, *catalase2* (*cat2*) and *wrky25/cat2* double-knock-out plants (**Figure S8**). As expected, *cat2* and *wrky25* had lower H_2_O_2_ scavenging capacity than wildtype plants resulting in a higher H_2_O_2_ content in the mutant lines (**Figures S8B, C**). Remarkably*, wrky25/cat2* double mutants showed an additive effect indicating that higher H_2_O_2_ content in *wrky25* mutant plants is not due to lower catalase activity. This was also visualized by the CAT-activity staining of a native PAGE, in which CAT2 activity of wildtype and *wrky25* mutant plants appear to be very similar (**Figure S8A**). Moreover, dark-induced senescence was more pronounced in *wrky25* or *cat2* mutant compared to wildtype leaves and was enhanced in the *wrky25/cat2* double mutant, correlating with their intracellular H_2_O_2_ contents (**Figures S8B, D**).

### WRKY25 Enhances *WRKY53* Response to Oxidizing Conditions

**Figure 7:**
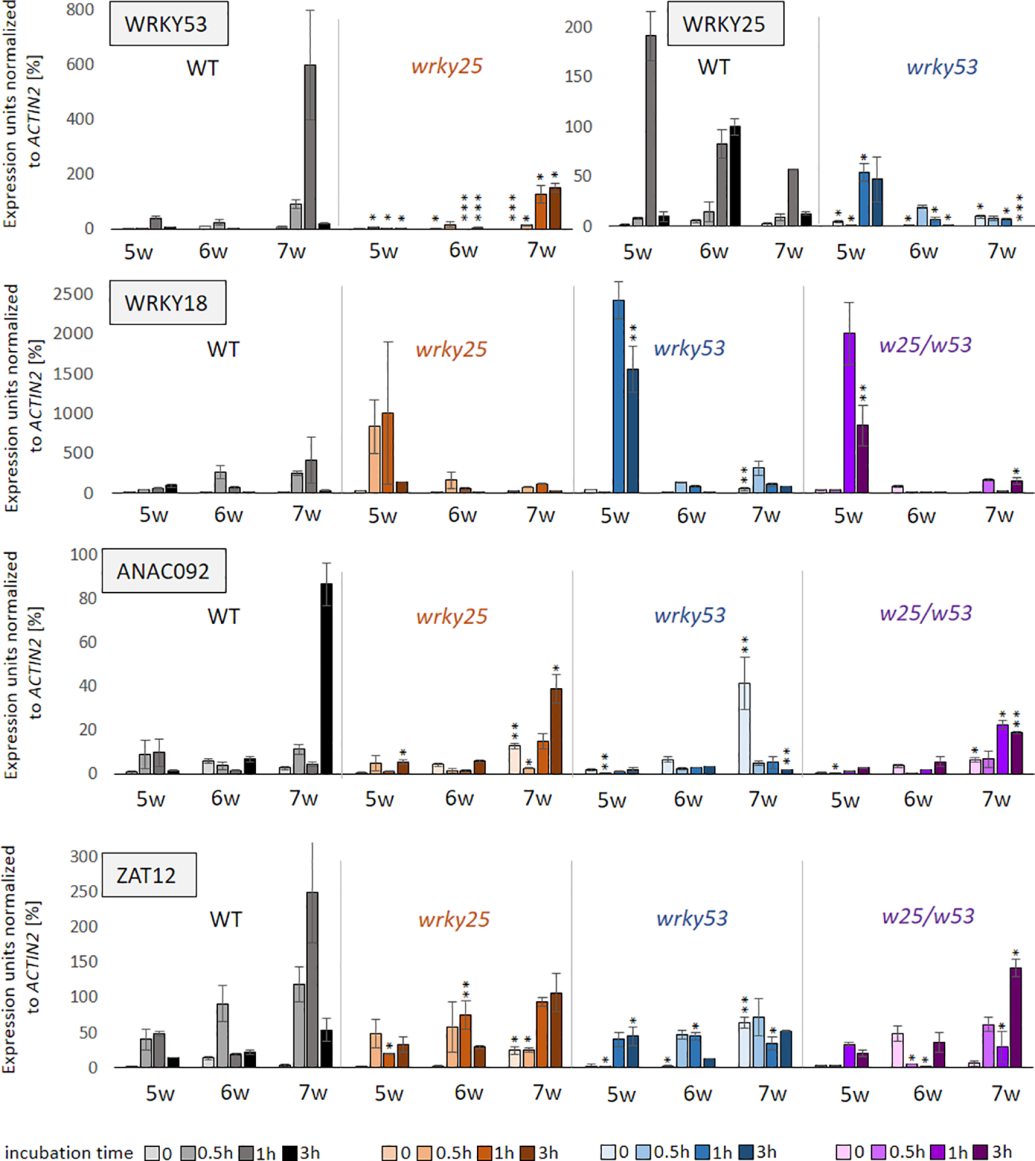
Influence of oxidizing conditions on gene expression. Leaves of position 10 were harvested of six plants of Col-0 (WT), *wrky25* (orange) and *wrky53* (blue) single and double (purple) mutant plants, respectively. Leaves of 5, 6 and 7 week-old-plants were incubated in 10 mM H_2_O_2_ for 0–3 h. After incubation, three leaves were pooled and expression of *WRKY18, WRKY25*, *WRKY53*, *ZAT12* and *ANAC092* was determined by qRT-PCR in these two pools. Expression values normalized to *ACTIN2* expression are presented (mean values ± SD, n = 2, one biological replicate consists already of a pool of three plants for each timepoint). Kruskal–Wallis-test was performed for statistically significant differences of all values at each timepoint compared to Col-0 (*P ≤ 0.05, **P ≤ 0.01, ***P ≤ 0.001).

Because *WRKY53* is strongly up-regulated after treatment with H_2_O_2_ in Arabidopsis (Miao et al., 2004; Xie et al., 2014), WRKY25 DNA-binding is redox-sensitive (**Figure 2A**) and its positive effect on *WRKY53* expression is diminished under oxidizing conditions (**Figure 3B**), we wanted to find out, whether WRKY25 is required for the induction of *WRKY53* expression after H_2_O_2_ treatment. Therefore, leaves of *wrky25* and *wrky53* single as well as double mutants and WT plants were detached and incubated in 10 mM H_2_O_2._ The expression of *WRKY53* and several other H_2_O_2_-responsive genes (*WRKY25*, *ANAC092, WRKY18* and *ZAT12*) was determined after 0 min, 30 min, 1 h and 3 h using qRT-PCR (**Figure 7**). All tested genes were responsive to H_2_O_2_ in wildtype. *WRKY53* expression increased most prominently in 7-week-old plants after 1 h of H_2_O_2_ treatment. This response is clearly dampened in *wrky25* mutant leaves. In contrast, the *WRKY25* mRNA level highly increased in leaves of young 5-week-old wildtype plants 1 h after H_2_O_2_ treatment and responsiveness becomes lower with age. Again, this response is diminished in *wrky53* mutant leaves in all tested developmental stages indicating that WRKY25 is involved in H_2_O_2_ response of *WRKY53* and *vice versa*. *ANAC092* responded most prominent also in 7-week-old leaves, similar to *WRKY53*. This response is also suppressed in the *wrky25* and in the *wrky53* leaves, and even more in the double mutant suggesting that both factors are involved in the H_2_O_2_ responsiveness of *ANAC092*. The same held true for *ZAT12* expression, here a higher basal expression could be observed in 7-week-old leaves of *wrky53* so that the H_2_O_2_ treatment did not lead to a further induction. In contrast, induction of *WRKY18* expression by H_2_O_2_ was much more pronounced in 5-week-old *wrky25*, *wrky53* and the double mutant compared to wildtype leaves, whereas the response was attenuated in older stages in all mutant lines. This supports the idea of a variable function of WRKY53 and WRKY25 on the *WRKY18* promoter: in early developmental stages, they act as repressors, in later stages as activators. Taken together, WRKY25 as well as WRKY53 are involved in H_2_O_2_ induction of variable genes including each other and, depending on the developmental stage of the plants; they can have opposing effects on the same gene promoters, again indicating a very complex regulatory interaction.

## Discussion

ROS, especially hydrogen peroxide, act as signaling molecules during senescence and/or stress responses. However, how this signal is perceived and transmitted into senescence onset and progression or stress response activation is still far from being understood. One of the central features of senescence is a massive change in the transcriptome, in which photosynthesis related genes are shut down and genes related to degradation and remobilization processes are turned on. Therefore, transcription factors would be ideal candidates to take up ROS signals directly. Indeed, for some transcription factors of different families such as class I TCP factors (Viola et al., 2013), HSF8 (Giesgut et al., 2015) or the bZIP factor GBF1 (Shaikhali et al., 2012), a redox-sensitive action has already been disclosed. Moreover, the plant specific protein GIP1 enhances DNA-binding activity of GBF3 and reduces DNA-binding activity of other members of the G-group bZIP factors in Arabidopsis, namely bZIP16, bZIP68, and GBF1, under non-reducing conditions through direct physical interaction. Whereas reduced GIP1 predominantly exists in a monomeric form and is involved in formation of DNA–protein complexes of G-group bZIPs, oxidized GIP1 is released from these complexes and instead performs chaperone function (Shaikhali, 2015). Due to space limitation, not all examples can be mentioned here, but taken together redox conditions can influence gene expression through the action of transcription factors in several ways: changing DNA-binding activity or activation potential or intracellular localization or interaction with specific partners or proteolytic degradation or a combination of those. He et al. (2018) just recently reviewed this topic very nicely. Here, we could show that WRKY25 DNA-binding activity is redox-sensitive, and that these redox-sensitive changes in activity are reversible as a function of the redox conditions (**Figure 2**). Not all WRKY factors show these features, as e.g. WRKY18 DNA-binding activity appears not to be redox-sensitive at all and WRKY53 DNA-binding activity was only very slightly influenced by changes in the redox environment (**Figure S2**). Redox sensitivity often relies on the alteration of the redox state of certain Cys residues. In WRKY25 belonging to the group I WRKY factors, two DNA-binding domains including CX_4-5_CX_22-23_HXH zinc fingers are present. Moreover, an additional Cys can be found very closely to the N terminus, which cannot be detected in WRKY53 or WRKY18 proteins and could therefore be involved in redox sensitivity. Whereas WRKY53 has no other Cys besides the Cys of the zinc finger, WRKY18 has three additional Cys residues but is not redox sensitive at all (**Figure S9**). Hence, we speculate that either the two zinc fingers are necessary to confer redox sensitivity or an additional Cys has to be at a certain position within the protein to contribute to redox sensitivity. However, this will be subject of further investigations. Currently, we are mutating the additional Cys in WRKY25 to see whether this residue is involved or responsible for the redox sensitivity of WRKY25. Moreover, we will include an additional Cys at the N terminus of WRKY18 to see whether we can render WRKY18 redox sensitive. In contrast to WRKY18, which strongly binds to all W-boxes of the *WRKY53* promoter (Potschin et al., 2014), WRKY25 binds selectively to a specific W-box in the promoter of *WRKY53* and can positively influence its expression (**Figures 1** and **3**). Under oxidizing conditions, activation of *WRKY53* expression by WRKY25 is dampened (**Figure 3**). Even though binding and transactivation is lower under oxidizing conditions, WRKY25 is still involved in the response of the WRKY53 promoter to oxidizing conditions *in planta*, as H_2_O_2_ response of *WRKY53* was much lower in the *wrky25* mutants compared to WT plants, especially in later stages (**Figure 7**). At the first glance, this appears to be a contradiction, but as WRKY25 is also involved in down-regulation of H_2_O_2_ contents and negatively regulates its own expression, two negative feedback loops are at work. This indicates that WRKY25 function might be to prevent an overshooting of the reaction to H_2_O_2_. In addition, not only *WRKY53* response to oxidative stress appears to be attenuated by WRKY25 but also *ZAT12* and *ANAC092* response. In contrast, *WRKY18* reaction appears to be enhanced, but only in young plants (**Figure 7**). As already mentioned before, *WRKY25* expression is induced by H_2_O_2_, whereas WRKY25 at the same time reduces intracellular H_2_O_2_ contents, especially in later stages of senescence, as lower or higher H_2_O_2_ levels were measured in *WRKY25* overexpressing plants and *wrky25* mutant or knock-down lines, respectively (**Figures 6** and **7**). High H_2_O_2_ contents in later stages of senescence are most likely involved in membrane deterioration and lipid peroxidation processes as part of the senescence degradation processes (Chia et al., 1981). This is in line with the senescence acceleration or delay of the *WRKY25* overexpressing plants and *wrky25* mutant or knock-down lines, respectively (**Figure 4**, **Figure S8**).

A simple gene for gene relationship between *WRKY25* and *WRKY53* would suggest opposite phenotypes. As *WRKY53* has been characterized as positive regulator of leaf senescence (Miao et al., 2004), overexpression of *WRKY25* should lead to increased *WRKY53* levels and to the same senescence phenotype as *WRKY53* overexpression. *Vice versa*, knock-down or mutation of *WRKY25* should exhibit the same phenotype as knock-down or mutation of *WRKY53*. However, the senescence phenotype of *WRKY25* overexpressing plants and *wrky25* mutant or knock-down lines was found to be exactly opposite to the expected phenotype of a positive *WRKY53* regulator. This can be explained by the fact that WRKY25 and WRKY53 are not acting in a simple signal transduction pathway but in a complex regulatory network between many members of the WRKY transcription factor family showing multilayer feedback regulations. In the same line of evidence, WRKY18 was characterized to be a negative up-stream regulator as well as a down-stream target and a protein interaction partner of WRKY53 (Potschin et al., 2014). Here, we could show that WRKY25 is also a redox sensitive up-stream regulator and down-stream target gene of WRKY53 (**Figures 3** and **7**). Moreover, WRKY25 appears to be involved in the H_2_O_2_ response of WRKY18 and WRKY53 expression but in opposite directions and at different times (**Figure 7**). In addition, MEKK1 action brings in a further layer of complexity. Co-expression of MEKK1 led to a reversal of WRKY18 action on *WRKY53* expression, since a 35S:*MEKK1* construct as co-effector to 35S:*WRKY18* reversed the repressor function of WRKY18 on the *WRKY53* promoter to an activator (**Figure S4A**). In contrast, the activator function of WRKY25 on the expression of the *WRKY53* promoter is enhanced approx. 2-fold by the addition of 35S:*MEKK1* as co-effector construct (**Figure 3C**). Whether this is due to a direct phosphorylation of WRKY25 by MEKK1, taking the same short cut as already shown for WRKY53 (Miao et al., 2007), or through classical MAPK signal transduction will be subject of further investigations. Noteworthy, WRKY25 and WRKY33 interact with many VQ proteins (Cheng et al., 2012), one of which is MKS1 (MAP KINASE SUBSTRATE 1), a substrate of MAPK4 (Andreasson et al., 2005). For WRKY33 it was shown that it exists in nuclear complexes with MPK4 and MKS1. Upon activation of MPK4 *via* MEKK1 and MKK1/2 signaling, MKS1 is phosphorylated by MPK4 and WRKY33 is released from MPK4 interaction and activates its downstream genes such as *PAD3* encoding an enzyme required for antimicrobial camalexin production (Qiu et al., 2008). Moreover, WRKY25 negatively regulates SA-mediated defense responses against *Pseudomonas syringae* (Zheng et al., 2007) and MPK4 is a repressor of SA-dependent defense responses (Petersen et al., 2000). Furthermore, MEKK1 kinase activity and protein stability is regulated by H_2_O_2_ in a proteasome-dependent manner and *mekk1* heterozygous mutants were compromised in ROS-induced MPK4 activation. Like WRKY25, MEKK1 regulates accumulation of intracellular H_2_O_2_ and alters expression of genes related to ROS signaling and homeostasis such as *ZAT12* (Nakagami et al., 2006). Like *WRKY25* and *WRKY53*, *MEKK1* expression is up-regulated by H_2_O_2_ treatment and mRNA levels start to increase with onset of senescence in parallel to *WRKY53* (Miao et al., 2007). Therefore, the influence of MEKK1 on the transactivation activity of WRKY25 provides another link to redox signaling. Moreover, we could show by conditional knock-down of *MEKK1* in plants that MEKK1 is part of the complex senescence regulation (**Figure S4**).

Expression of *WRKY25* is not only induced by oxidative stress but also during heat or salt stress. Moreover, *WRKY25* overexpressing plants were not only more tolerant to oxidative stress (**Figure 6**) but also to salt stress (Jiang and Deyholos, 2009) as well as to high temperatures (Li et al., 2011). During heat stress, WRKY25, WRKY26, and WRKY33 were positively cross-regulated, which confirms the complexity of the WRKY network (Li et al., 2011). Remarkably, ROS levels increase during salt and heat stress pointing to the possibility that *WRKY25* induction under salt and heat stress is mediated by oxidizing conditions. Many WRKY factors including WRKY25 and WRKY53 are up-regulated more than 5-fold in various plant lines with altered intracellular levels of specific ROS (Gadjev et al., 2006). In the same line of evidence, expression of *WRKY18*, *WRKY25* and *WRKY53* was also increased in *cat1,2,3* triple mutant plants (Su et al., 2018). Moreover, not only *WRKY25* gene expression and its DNA-binding activity are altered by higher ROS levels but WRKY25 is also involved in the regulation of the intracellular H_2_O_2_ content, especially in later stages of development (**Figure 6**) creating a feedback loop.

A further level of complexity is installed by epigenetic control of the WRKY gene expression. JMJ27, a jumonji-family demethylases, removes repressive H3K9me2 and H3K9me1 marks and thereby activates transcription. ChIP analysis revealed that the chromatin at the *WRKY25* promoter was hyper-methylated in *jmj27* mutants indicating that JMJ27 regulates *WRKY25* expression at least in part by directly controlling methylation levels of H3K9 histones (Dutta et al., 2017). *WRKY53* expression is also regulated by epigenetic changes in histone methylation (Ay et al., 2009). Moreover, the WRKY53 protein was detected in a complex with histone deacetylase 9 (HDA9) and POWERDRESS to recruit this complex to W-box containing promoter regions of key negative senescence regulators to remove H3 acetylation marks (Chen et al., 2016). Therefore, WRKY53 expression is regulated by epigenetic changes on its own promoter but the WRKY53 protein is also involved in changing epigenetic marks on other promoters.

**Figure 8:**
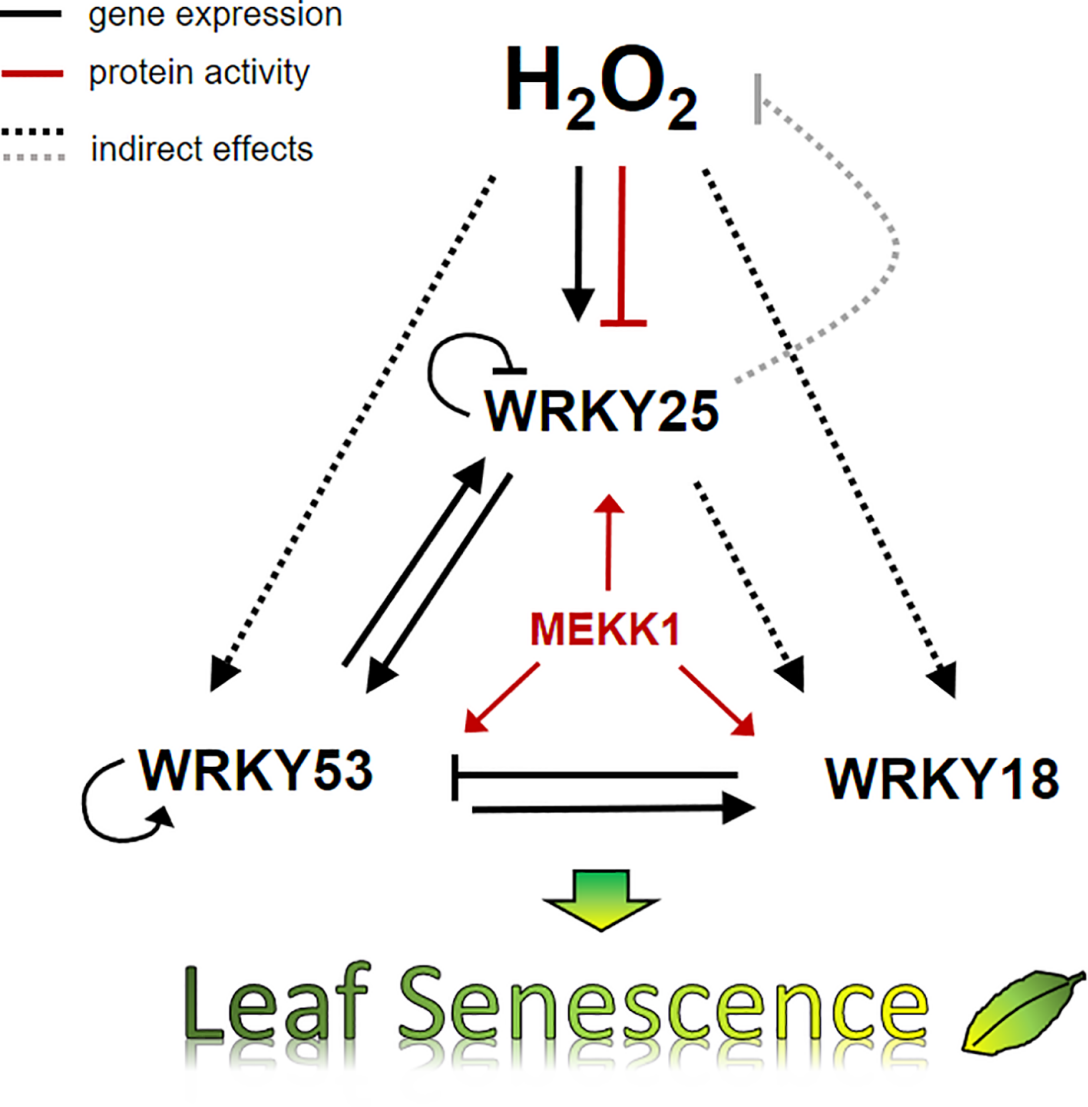
Model of H_2_O_2_ and the WRKY18-53-25 subnetwork. A model summarizing the impact of H_2_O_2_ and WRKY25 on senescence in 7 week-old plants is presented. Solid lines show direct interactions whereas dotted lines show interaction, which may be direct or indirect. Black arrows describe the effects on gene expression, red arrows effects on protein activity, and the grey line effects on the intracellular hydrogen peroxide level. The expression of all three WRKY genes of the small WRKY53-25-18 subnetwork are controlled by hydrogen peroxide contents and hydrogen peroxide has a direct negative effect on the binding activity of WRKY25 to DNA. All three genes are under feedback control of their own gene products. In addition, MEKK1 increases the activity of all three WRKY factors. Moreover, WRKY25 can form heterodimers with WRKY53 and the heterodimer has a lower transactivation activity compared to the WRKY25 homodimer. This interplay determines in the end whether leaf senescence is accelerated or delayed.

We have summarized our data in a model, which describes a small subnetwork between WRKY18, WRKY25 and WRK53 and the role of H_2_O_2_ in this subnetwork at the onset of senescence (**Figure 8**). Several feedback loops are installed to control an overshooting of the system and to supply a high plasticity, which is needed to constantly integrate all kinds of incoming intracellular and environmental signals. The complex interactions within this subnetwork of just three WRKY factors illustrates the high complexity of the whole WRKY network, which is not only regulated by H_2_O_2_ as signaling molecule but also highly controlled by salicylic and jasmonic acid. Moreover, the WRKY network is just a subsection of the higher order regulatory network of leaf senescence. Nevertheless, understanding the regulation of single components or subnetworks will in the long run help to decipher the different mechanisms acting in the whole network and contribute to modeling approaches.

## Materials and Methods

### Protein Expression and Extraction for DPI-ELISA

For protein expression of WRKY25 and WRKY53 in the *E. coli* strain BL21-SI, the coding sequences of *WRKY25* (1,182 bp) and *WRKY53* (975 bp) were cloned into the vector pETG-10A to be coupled with an N-terminal fused 6×His-tag. The *E. coli* cells were grown in 10 ml selective medium overnight. One hundred milliliter LB-medium were inoculated with 3 ml of this pre-culture and, after shaking for 1.5 h at 37°C, a final concentration of 1 mM IPTG was added for induction of protein expression. After 1 h of shaking at 18°C, cells were harvested (2,500 g, 20 min, 4°C) and suspended in protein extraction buffer (4 mM Hepes pH 7.5, 100 mM KCl, 8% (v/v) glycerol, 1× complete proteinase inhibitor (*Roche*) without EDTA). Proteins were extracted by sonication to keep native conditions. The protein concentrations of the crude extracts were detected by Bradford assays (Bradford, 1976; *Bio-Rad*).

### DPI-ELISA

The ELISA-based DNA–protein interaction assay was performed as described by Potschin et al. (2014). In brief, the 5' biotinylated double-stranded oligonucleotides were added to streptavidin-coated ELISA plates (*Thermo Scientific*). After blocking the plate with blocking solution *(Roche, blocking reagent for ELISA)*, crude extracts were diluted with protein dilution buffer (4 mM Hepes pH 7.5, 100 mM KCl, 8% (v/v) glycerol) and increasing protein concentrations (5, 10, 25 µg) were added to the DNA bound to the plates. The plates were incubated 1 h with mild shaking so that the biotinylated DNA–protein complexes were formed. Subsequently, wells were washed at least twice (*Qiagen blocking buffer*, *Anti-His-HRP conjugate kit*) and incubated for another hour with Anti His-HRP conjugate antibodies (*Qiagen*) diluted 1:1,500. After washing several times, positive interactions were detected by a peroxidase reaction with ortho-phenylenediamine (OPD-tablets, *Thermo Scientific*). The yellow color was measured using a plate-reader (*TECAN*, Safire XFluor4). For Redox-DPI-ELISAs, 25 µg of the protein crude extracts were used. Reduction or oxidation of the protein extracts was performed by adding either DTT or H_2_O_2_ (final concentration 5 mM). In order to show reversibility of the redox effects, a fraction of the DTT-reduced proteins was oxidized again by addition of increasing amounts of H_2_O_2_ (final concentration 5, 10 and 20 mM). Similarly, the oxidized proteins were reduced again by adding increasing amounts of DTT. After these redox-treatments, a DPI-ELISA was performed as described above. To conserve the redox-state of the proteins bound to the biotinylated DNA, the same DTT or H_2_O_2_ concentrations were added to the washing buffer and the antibody solution. The antibody reaction was not altered by these treatments.

### Protoplast Preparation and Transformation

Protoplasts derived from a cell culture of *A. thaliana* var. Columbia 0 were prepared following a standard protocol (for details see http://www.zmbp.uni-tuebingen.de/c-facilit/plant-transformation.html). These protoplasts were then treated with PEG1500 and transiently transformed with different constructs following the protocol published in Mehlhorn et al. (2018). Effector and reporter constructs were co-transfected with a luciferase construct for GUS reporter assays (for details see *GUS Reporter Assay*).

### GUS Reporter Assay

Arabidopsis protoplasts were transformed using 5 µg of effector and 5 µg of reporter plasmid DNA. As an internal transformation control, a luciferase construct (pBT8-35SLUCm3) was co-transfected. After incubation overnight in the dark, GUS activity assays were performed with the protoplast as described by Jefferson et al. (1987). To correct for transformation efficiency, GUS activity was normalized to luciferase fluorescence. As effector constructs, the coding sequences of *WRKY25* (1182 bp), *WRKY53* (975 bp) and *MEKK1* (1827 bp) cloned into the vector pJAN33 were used. As reporter construct, a 3,000-bp-fragment upstream of the *WRKY25* start codon and a 2,759-bp-sequence upstream of the start codon of *WRKY53* was cloned into the binary vector pBGWFS7.0. The 3'-AT (3-Amino-1,2,4-triazole) GUS assays were performed as described above except that 10 mM 3'-AT or the same volume of water was added before overnight incubation.

### Plant Material and Cultivation

All experiments were performed with *A. thaliana* Ecotype Columbia 0 (Col-0). Plants were grown on standard soil under long day conditions (16 h of light) with only moderate light intensity (60–100 μmol s^−1^ m^−2^) in a climatic chamber at 20°C (day) and 18°C (night). Bolts and flowers developed within approx. 4–5 weeks. Individual leaf positions within the rosettes were coded with different colored threads, so that individual leaves could be analyzed according to their age even at very late stages of development (Hinderhofer and Zentgraf, 2001; Bresson et al., 2018). Numbering started with No. 1 for the first true leaf without taking the cotelydones into account. Plant material was harvested always at the same time to avoid circadian effects. The Nottingham Arabidopsis Stock Centre (NASC) kindly provided the T-DNA insertion line of *WRKY25* (SAIL_529_B11; previously characterized in Jiang and Deyholos, 2009), of *WRKY53* (SALK_034157, previously characterized in Miao et al., 2004), and *CAT2* (SALK_057998, previously described in Queval et al., 2007). Using PCR, homozygous plants were characterized with different combinations of gene specific and T-DNA left border primers. Double-knock-out mutants (*wrky25/wrky53*) were generated by crossing *wrky25* and *wrky53* mutants. F2 progenies were selected for homozygous double-knock-out plants by PCR. Dr. Changle Ma, Shangdong Normal University, China (Su et al., 2018), kindly provided seeds of the homozygous *wrky25/cat2* double-knock-out plants. The *WRKY25* overexpressing plants were transformed by floral dip of Col-0 wildtype plants into *Agrobacterium tumefaciens* cultures in two different attempts. First, a 35S:*WRKY25* construct was transformed leading to plants in which the transgene induced gene silencing (plant line 35S:*W25si*; pB7RWG2) and, therefore, was used as a *knock-down* line. Second, a *UBQ10:WRKY25* construct was transformed (plant line UBI:*W25-1* and UBI:*W25-2*; pUBN-GFP-Dest) and overexpression was confirmed by qRT-PCR. For the germination experiments, seeds of different plant lines were sterilized by sodium hypochlorite and plated on ½ MS-plates (1 L: 2.15 g MS micro and macro elements (Duchefa), 15 g sucrose, pH 5.7–5.8, 8 g agar).

### Senescence Phenotyping

For the evaluation of leaf senescence phenotypes, rosette leaves were aligned according to the age of the leaves with the help of a color code and a variety of parameters indicating the state of senescence were measured (Bresson et al., 2018). Leaves of six plants per timepoint were analyzed. At position 5 and 10, Fv/Fm values were determined using the Imaging-Pulse-Amplitude-Modulation (PAM) method, indicating the activity of the photosystem II (PSII) (Chlorophyll fluorometer Maxi version; ver. 2-46i, Walz GmbH, Effeltrich, Germany). Leaves were photographed according to their age and by an automated colorimetric assay (ACA) pixelwise grouped into four categories: green leaves (green), leaves starting to get yellow (green-yellow), completely yellow leaves (yellow) and brown and/or dead leaves (brown/dead). (ACA; Bresson et al., 2018; http://www.zmbp.uni-tuebingen.de/gen-genetics/research-groups/zentgraf/resources.html)

In addition, RNA was extracted from leaves No. 6 and 7 and qRT-PCR analyses were performed for the senescence-associated marker genes *ANAC092* (At5g39610) encoding a NAC-domain transcription factor, *CAB1* (At1g29930) encoding a subunit of light-harvesting complex II, *SAG12* (At5g45890) encoding a cysteine protease and different WRKY genes (*WRKY53* (At4g23810), *WRKY18* (At4g31800), *WRKY40* (At1g80840)). Expression was normalized to *ACTIN2* (At3g18780).

### Quantitative RT-PCR

Total RNA was extracted with the RNeasy Plant Mini Kit (*Qiagen*). Subsequent cDNA synthesis was performed with RevertAid Reverse Transcriptase (*Thermo Scientific*. For the qRT-PCR, KAPA SYBR^®^ Fast Biorad iCycler (*KAPA Biosystems*) master mix was used following the manufacturer´s protocol. Expression of analyzed genes was normalized to *ACTIN2*. In order to keep the results comparable to former results, we only used *ACTIN2* as reference gene since *ACTIN2* has been proven to be very stably expressed all over leaf development (Panchuck et al., 2005). Relative quantification to *ACTIN2* was calculated with the ΔΔCT-method according to Pfaffl (2001). Primers and Atg numbers are indicated in **Table S1**.

### H_2_O_2_ Measurement and Treatments

For oxidative stress treatment during germination, 10 mM H_2_O_2_ was added to the 1/2 MS agar (1 L: 2.15 g MS micro and macro elements (*Duchefa*), 15 g sucrose, pH 5.7–5.8, 8 g agar) and seeds were spread on plates with and without H_2_O_2_. After 3 days in darkness, the plates were incubated in light in the climate chambers and the number of green seedlings was counted after 7–10 days. This experiment was repeated six times, plates were photographed, green seedlings counted and summarized according to their tolerance against H_2_O_2_ in a heat map.

For intracellular H_2_O_2_ measurement, carboxy-H_2_DCFDA (2',7'-Dichlordihydrofluorescein-diacetat) was chosen, which is able to passively diffuse across cellular membranes as non-polar dye. After deacetylation by an intracellular esterase, the molecule gets polar and is trapped inside the cells. The deacetylated carboxy-H_2_DCFDA can then be oxidized by H_2_O_2_ and converted to the highly fluorescent di-chlorofluorescein (DCF). Therefore, only intracellular H_2_O_2_ is measured. Leaves of position 8 of 4- to 8-week-old plants were harvested and incubated for exactly 45 min in carboxy-H_2_DCFDA working-solution (200 µg in 40 ml MS-Medium pH 5.7–5.8). Subsequently, the leaves were rinsed with water and frozen in liquid nitrogen. After homogenization in 500 µl 40 mM Tris pH 7.0, the samples were centrifuged at 4°C for 15 min. Fluorescence of the supernatant was measured in a *Berthold* TriStar LB941 plate reader (480 nm excitation, 525 nm emission).

For testing the response to H_2_O_2_ treatment, leaves of position 8 of 5, 6 and 7-week-old plants were incubated for 0, 30 min, 1 h and 3 h in 10 mM H_2_O_2_ including 0.1% Tween. After incubation time, leaves were washed in water and immediately frozen in liquid nitrogen. Gene expression was determined by qRT-PCR.

The decomposition of H_2_O_2_ was examined using commercially available peroxide strips (Dosatest peroxide test strips 100, VWR Chemicals). Therefore, leaf discs were excelled of leaves of position 5 of 6- and 7-week-old plants and were incubated into a 30 mg/l H_2_O_2_ solution. Strips were submerged for 1 s into the solution immediately after placing the leaf disc into the solution (timepoint 0 min) and again after 2 h. The amount of peroxide can be read out by the given control color scale. The weaker the blue color the less peroxide is present in the solution.

## Data Availability Statement

All datasets generated for this study are included in the article/**Supplementary Material**.

## Author Contributions

Conceptualization was done by UZ, MM and JD. Methodology was developed by MM and JD. Experiments were performed by JD, MM, LR, SN and H-CL. Data and formal analysis was done by JD, JB and LR. The original draft was written by UZ. Reviewing and editing was done by JD, LR, MM and UZ. Funding acquisition: UZ. Supervision: UZ.

## Funding

This work was financially supported by the Deutsche Forschungsgemeinschaft (DFG), Collaborative Research Centre 1101 (SFB1101, TP B06). The Alexander v. Humboldt Foundation supported JB.

## Conflict of Interest

The authors declare that the research was conducted in the absence of any commercial or financial relationships that could be construed as a potential conflict of interest.
