# Supplementary material for: Arabidopsis thaliana WRKY25 Transcription Factor Mediates Oxidative Stress Tolerance and Regulates Senescence in a Redox-Dependent Manner
Source: Front Plant Sci. 2020 Jan 23;10:1734. doi: 10.3389/fpls.2019.01734 (PMC6989604; doi:10.3389/fpls.2019.01734)
Supplement: Supplementary file 1 [file Presentation_1.pdf]

Arabidopsis thaliana WRKY25 transcription factor mediates oxidative stress tolerance and regulates senescence in a redox-dependent manner.

Jasmin Doll[#], Maren Muth[#], Lena Riester, Sabrina Nebel, Justine Bresson, Hsin-Chieh Lee, Ulrike Zentgraf

Center for Plant Molecular Biology (ZMBP), University of Tuebingen, Auf der Morgenstelle 32, 72076 Tuebingen, Germany

[#]shared first author

Supplementary Material

Figures:
S1: WRKY protein expression in *E. coli*
S2: Redox-DPI-ELISA with WRKY18 and WRKY53 protein
S3: 3´-AT treatment of protoplasts provokes an oxidative cell status
S4: Role of MEKK1 in WRKY18 driven *WRKY53* expression and senescence
S5: *WRKY25* expression analysis for lines with altered *WRKY25* expression
S6: Comparison of plant development
S7: Fv/Fm ratio measurements
S8: Dark-induced senescence and antioxidative capacity of *wrky25*, *cat2* and *wrky25/cat2*
S9: Cys residues in WRKY25, WRKY18 and WRKY53

Tables:
S1: Oligonucleotides for qRT-PCR

Methods related to supplemental Figures:
SM1: Dark-induced senescence phenotyping
SM2: Cell-free GUS reaction
SM3: H_2O_2 content in Arabidopsis protoplasts
SM4: Catalase zymograms

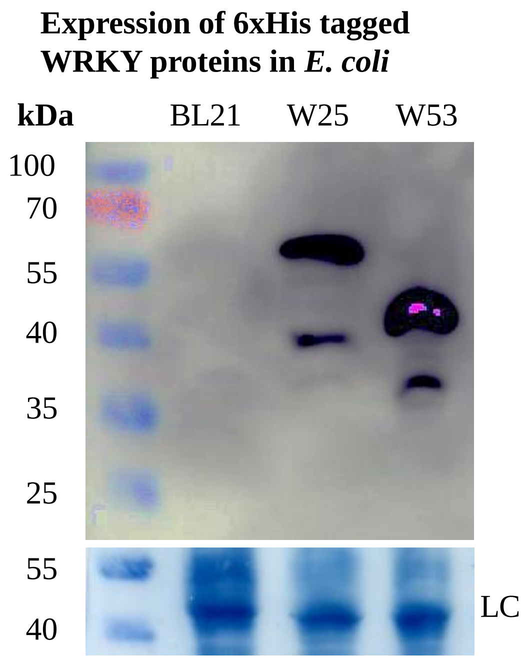

Figure S1: WRKY protein expression in *E. coli*.
Crude extracts of empty *E. coli* BL21 cells and BL21 cells expressing 6xHis-tagged WRKY25 or WRKY53 were prepared under native conditions and 25 µg of the raw lysates were loaded on the SDS PAGE. After blotting, the PVDF membrane was detected using Penta-His-HRP conjugate kit (*Qiagen*, 1:1500). After antibody detection, the PVDF membrane was stained with amido black as loading control (LC).

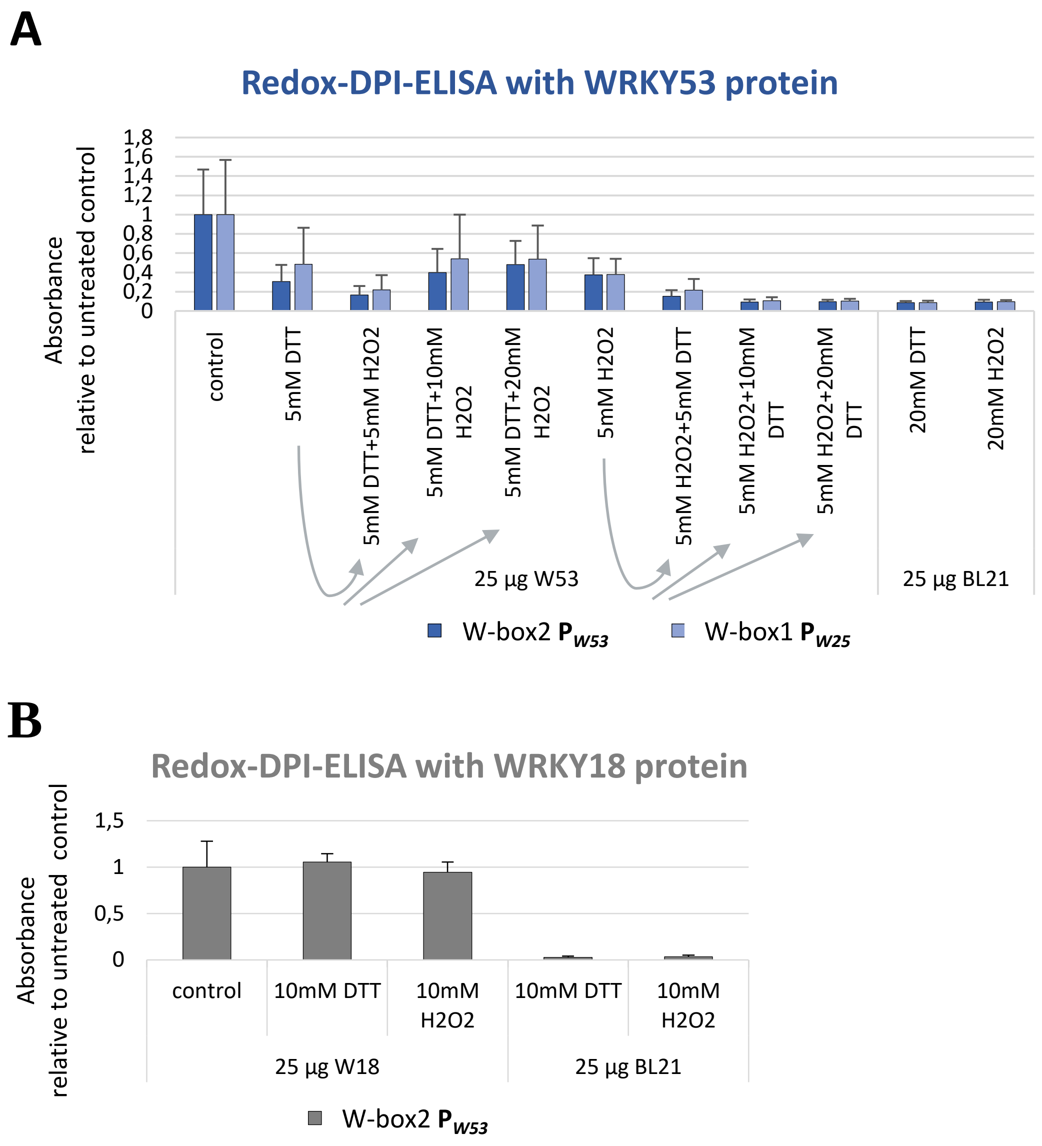

Figure S2: Redox-DPI-ELISA with WRKY18 and WRKY53 protein.

A. Redox-DPI-ELISA with 25 µg of crude extracts of *E. coli* BL21 cells expressing WRKY53 proteins and the 5'biotinylated annealed oligonucleotides W-box2 P_{W53} and W-box1 P_{W25}. Absorbance values are indicated relative to control without treatment (mean values + SD, n = 3). **B.** Redox-DPI-ELISA with 25 µg of crude extracts of *E. coli* BL21 cells expressing WRKY18 proteins and the 5'biotinylated annealed oligonucleotide W-box2 of the promoter of *WRKY53*. Protein extracts were reduced or oxidized by addition of either DTT or H_2O_2 to examine a redox-dependent binding. Absorbance values are indicated relative to control without treatment (mean values ±SD, n = 2). Kruskal-Wallis-test was performed for statistically significant differences (**P* ≤ 0.05). No significant differences were detected.

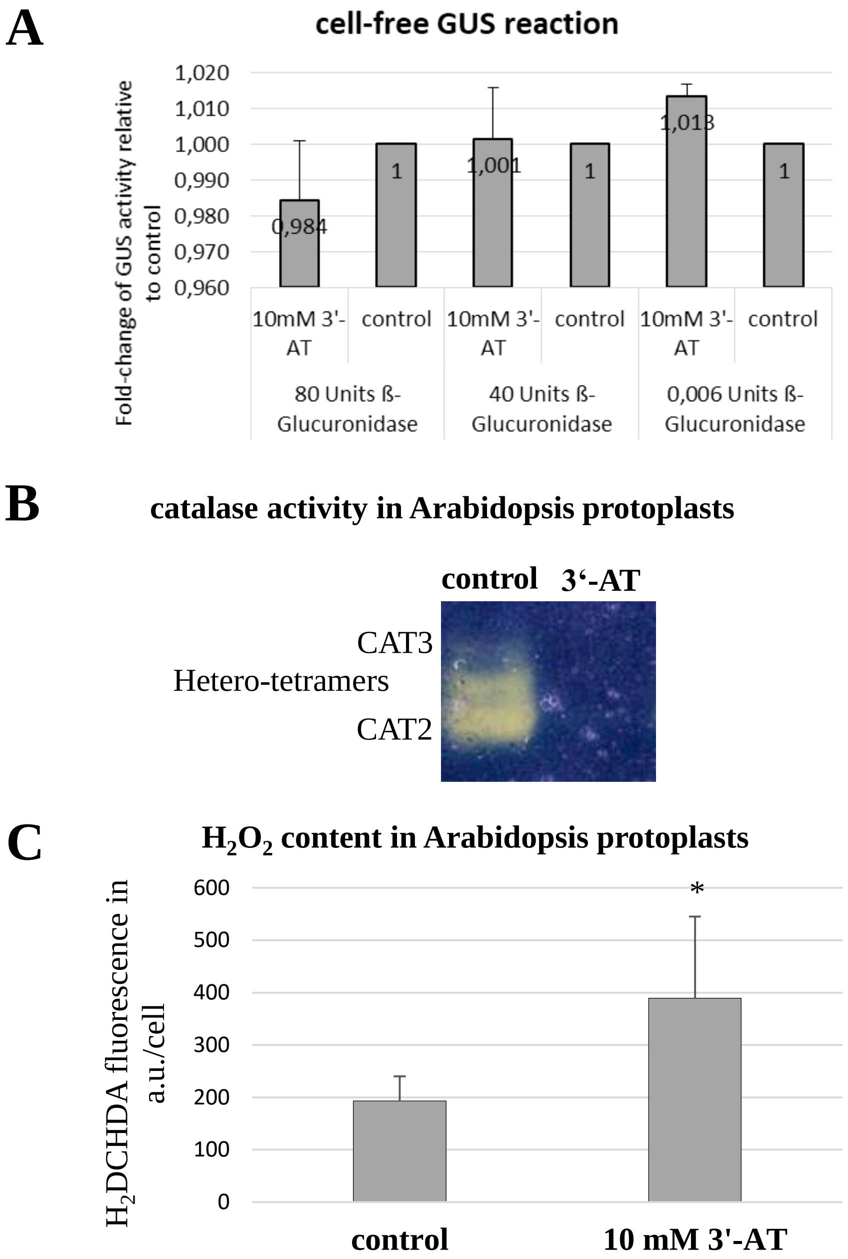

Figure S3: 3´-AT treatment of protoplasts provokes an oxidative cell status

(A) To prove that the 3’-AT treatment of the protoplasts has no influence on the GUS reaction itself, 10 mM 3’-AT or only buffer (control) was added to different concentrations of lyophilized ß-Glucuronidase (*Sigma-Aldrich*) and a normal GUS assay was performed. The values are presented relative to control (mean values ±SD, n = 3). **(B)** To show that the 3’-AT treatment of the protoplasts lowers the cellular catalase activity, 10 mM 3’-AT or water was added to isolated protoplasts overnight and catalase zymograms were performed. One example of a zymogram is presented (n = 3). **(C)** H_2O_2 content in Arabidopsis protoplasts was measured by using H_2DCFDA fluorescence; protoplasts were treated with 10 mM 3’-AT or water (control). The values are indicated relative to cell number (mean values ±SD, n = 5), Kruskal-Wallis-test was performed for statistical differences (*$P \leq 0.1$).

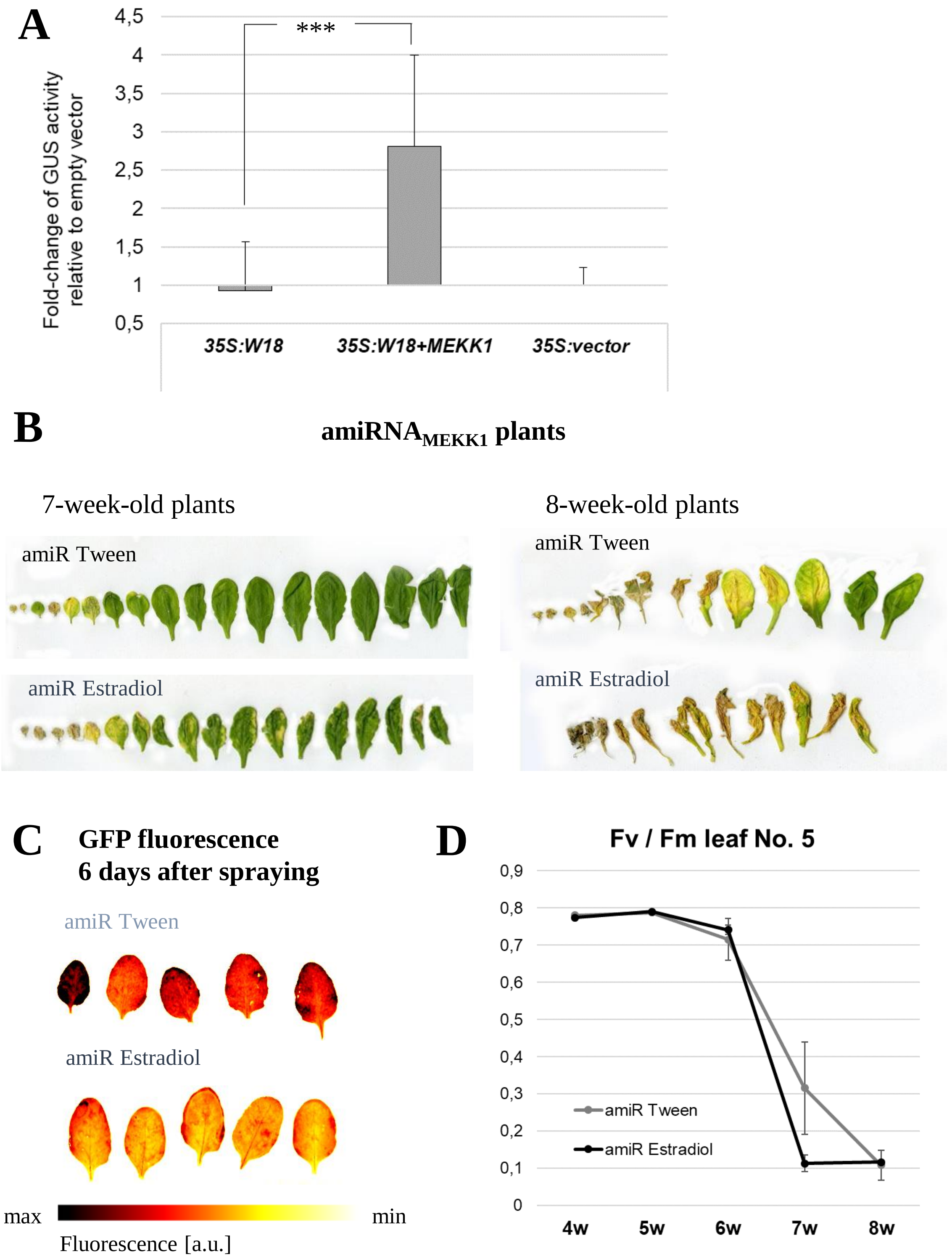

Figure S4: Role of MEKK1 in WRKY18 driven *WRKY53* expression and senescence.
(A) GUS-Assays were performed in *Arabidopsis* protoplasts using the *WRKY53* promoter and 35S:*WRKY18* and 35S:*MEKK1* as effector constructs. The empty vector was used as reference (mean values ±SE, n=4 independent transformations). **(B)** Rosette leaves of a ß-estradiol inducible amiRNA-MEKK1 line were arranged according to their age and representative pictures that show the senescence phenotype are presented. Plants were sprayed with 3 µM ß-estradiol; Tween-20 was used as a mock treatment. **(C)** The amiRNA-MEKK1 plants also contain a 35S:*GFP* fused to the target sequence of the amiRNA-MEKK1. Therefore, ß-estradiol induction of the amiRNA was assessed via the GFP fluorescence visualized with a Typhoon FLA 9500 laser scanner (*GE Healthcare*) 6 days after spraying (Li et al., 2013) **(D)** Fv/Fm values in leaf No. 5 were measured with the pulse amplitude modulation (PAM) method (mean values ±SE, n=5). Kruskal-Wallis-test was performed for statistical differences (*$P \leq 0.05$,**$P \leq 0.01$, ***$P \leq 0.001$).

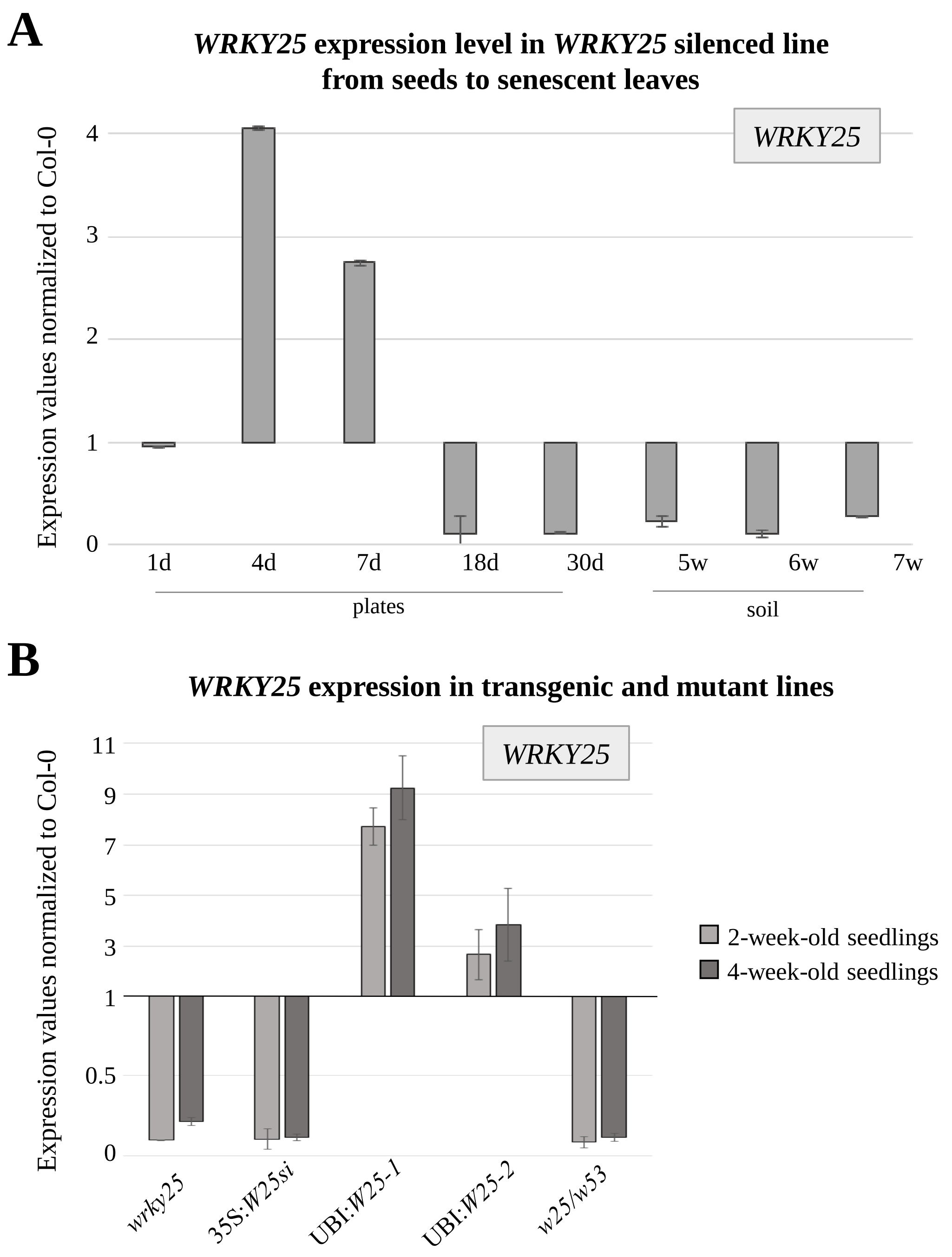

Figure S5: Gene expression analysis for lines with altered *WRKY25* expression. (A) The expression level of *WRKY25* was analyzed throughout the life span of the *WRKY25* silenced line (35S:*W25si*) from seeds to senescent leaves. For the timepoints 1 to 30 days seedlings grew on ½ MS media plates, timepoints 5 to 6 week old plants grew on soil. Two to three biological replicates were taken, each with more than 150 seedlings (1-7d), at least 15 seedlings (18-30d), and leaf No. 6 and 7 of 6 plants per timepoint (5-7w) were harvested. qRT-PCR values were normalized to the expression of the *ACTIN2* gene and the mean of values for Col-0 was set to 1 and values of the mutants were normalized to Col-0 (±SE). **(B)** Expression of *WRKY25* was analyzed in different *WRKY25* transgenic lines by qRT-PCR and normalized to the expression of the *ACTIN2* gene. Leaf material of at least 15 two-week-old seedlings or 4-week-old plants were harvested and pooled. The mean of values for Col-0 was set to 1 and values of the mutants were normalized to Col-0 (means of two technical replicates, ±SE).

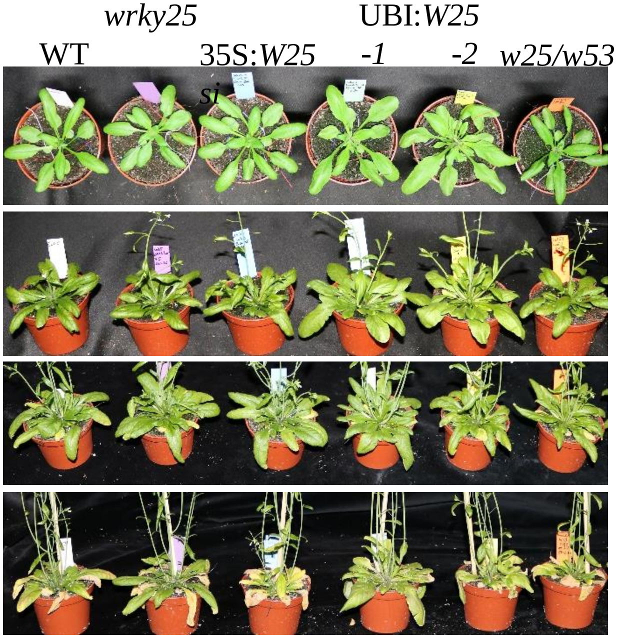

Figure S6: Comparison of plant development. Representative example of Col-0 (WT), *wrky25* mutant (*wrky25*), *WRKY25* overexpressing (UBI:*W25-1* and UBI:*W25-2*), *WRKY25* silenced (35S:*W25si*) and *wrky25-wrky53* double-knock-out-mutant (*w25/w53*) plants from week 5 to 8.

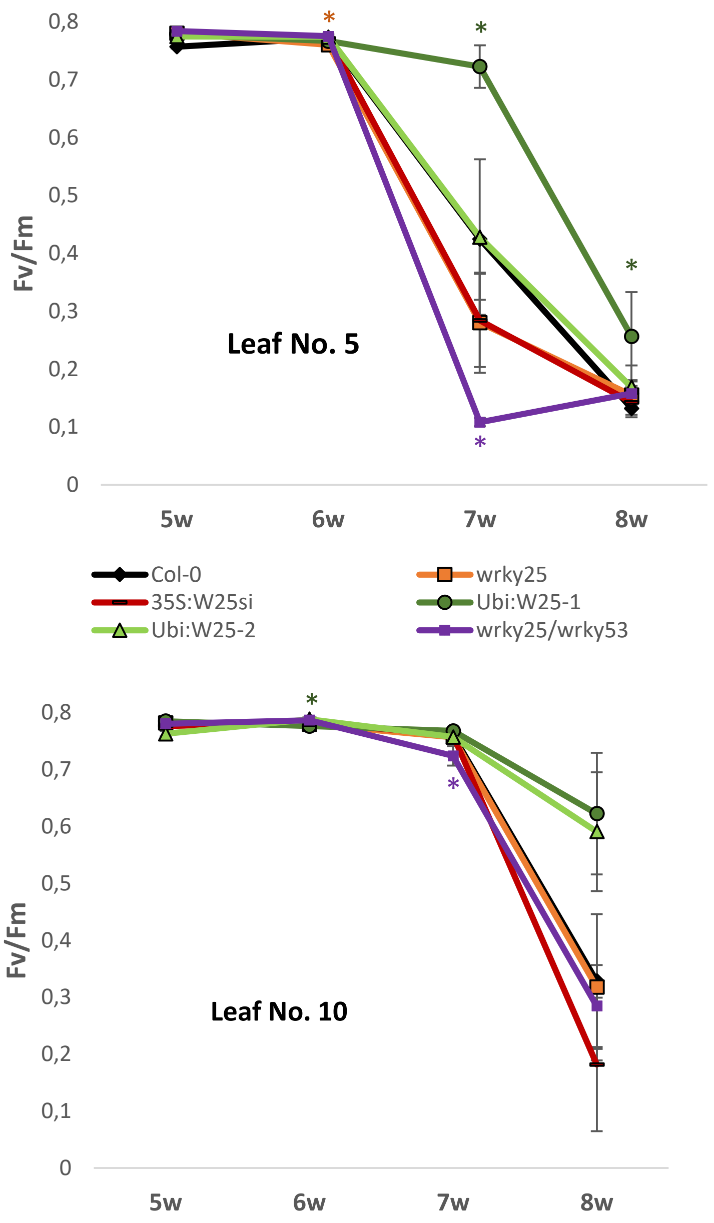

Figure S7: Fv/Fm ratio measurements.
Col-0 (WT), *wrky25* mutant (*wrky25*), *WRKY25* overexpressing (UBI:*W25-1* and UBI:*W25-2*), *WRKY25* silenced (35S:*W25si*) and *wrky25-wrky53* double-knock-out (*w25/w53*) plants were analyzed over development. Fv/Fm values were measured with PAM for leaves No. 5 and 10 for 5- to 8-week-old plants (mean values ±SE, n=6). Kruskal-Wallis-test was performed for statistical differences of all values compared to Col-0 (*$P \leq 0.05$).

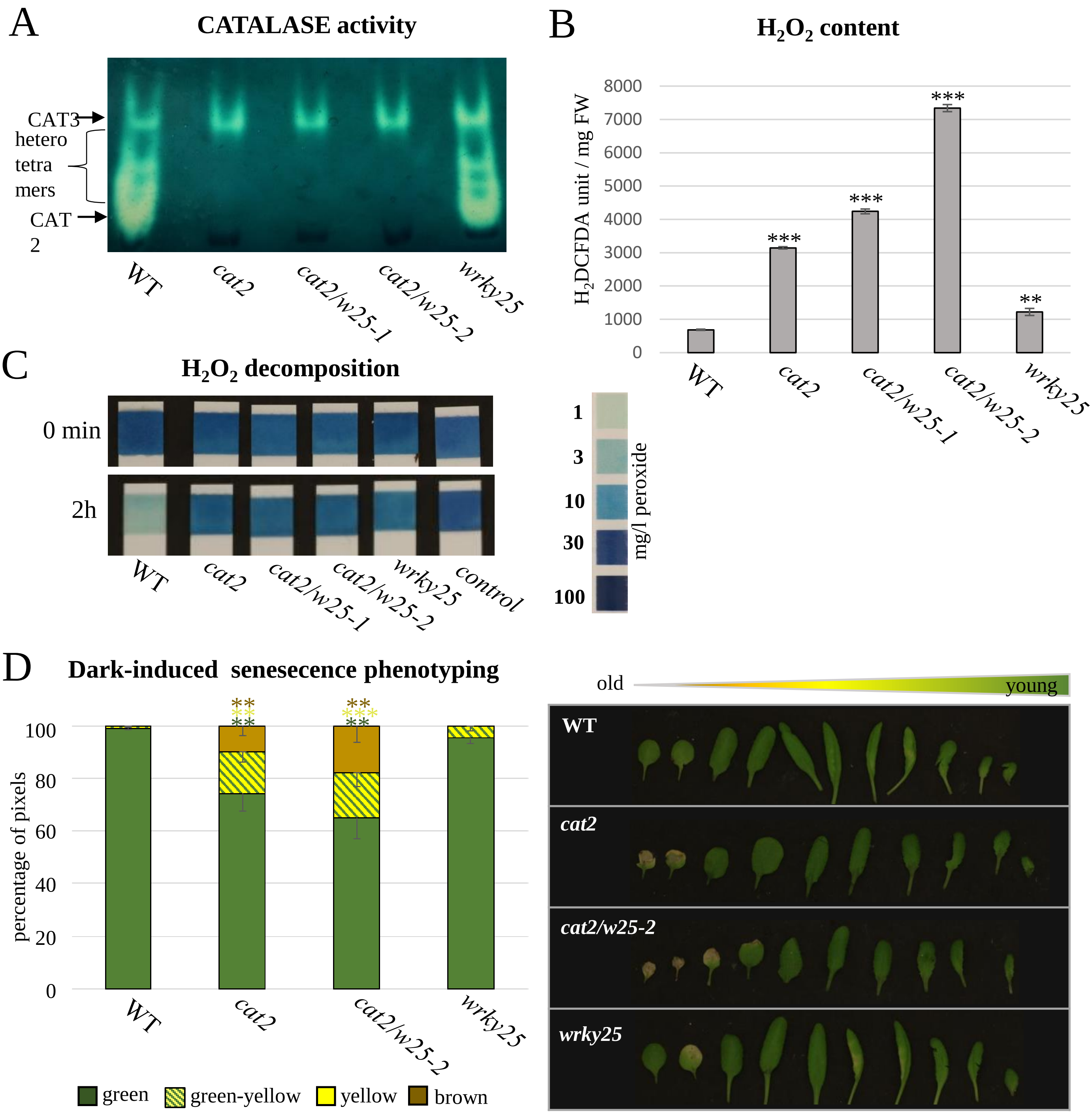

Figure S8: WRKY25 is involved in H_2O_2 clearance

(A) Protein crude extract was isolated from leaves of 7-week-old plants of Col-0 (WT), *wrky25* mutant (*wrky25*), *catalase2* mutant *(cat2)* and double-knock-out-mutant (*cat2/w25*) plants. Proteins were separated on a 7.5 % native gel and stained for catalase activity. Enzyme activity of CAT2 and CAT3 as well as the hetero-tetramers are visualized on a zymogram. **(B)** Intracellular H_2O_2 content was measured in leaves No. 8 of 6-week-old plants using the fluorescent dye H_2DCFDA. Values are normalized to mg freshweight (FW) (n=3, ±SE). **(C)** Decomposition of H_2O_2 was measured using commercially available peroxide strips. Two leaf discs of 7-week-old plants were incubated in 1 mM H_2O_2. As control H_2O_2 solution without leaf discs was measured. The peroxide content was measured immediately (0 min) and after 2 hours incubation (2h). **(D)** For dark-induced senescence, 4.5-week-old-plants were transferred into darkness for 7 days. A picture of the rosette leaves sorted according their age is shown. An automated colorimetric assay (ACA) categorizes the leaves into four groups according to their color (green, green/yellow, yellow, brown) and percentage of each category is depicted (mean values ±SE, n=3). Kruskal-Wallis-test was performed for statistically significant differences of all value compared to Col-0 (*$P \leq 0.05$, **$P \leq 0.01$, ***$P \leq 0.001$).

WRKY25 (group I)

1 MSSTSFTDLL GSSGVDCYED DEDLRVSGSS FGGYYPERTG SGLPKFKTAQ

51 PPPLPISQSS HNFTFSDYLD SPLLLSSSHS LISPTTGTFP LQGFNGTTNN

101 HSDFPWQLQS QPSNASSALQ ETYGVQDHEK KQEMIPNEIA TQNNNQSFGT

151 ERQIKIPAYM VSRNSNDGYG WRKYGQKQVK KSENPRSYFK CTYPDCVSKK

201 IVETASDGQI TEIIYKGGHN HPKPEFTKRP SQSSLPSSVN GRRLFNPASV

251 VSEPHDQSEN SSISFDYSDL EQKSFKSEYG EIDEEEEQPE MKRMKREGED

301 EGMSIEVSKG VKEPRVVVQT ISDIDVLIDG FRWRKYGQKV VKGNTNPRSY

351 YKCTFQGCGV KKQVERSAAD ERAVLTTYEG RHNHDIPTAL RRS

Zinc finger: $CX_{4-5}CX_{22-23}HXH$

WRKY18 (group IIa)

1 MDGSSFLDIS LDLNTNPFSA KLPKKEVSVL ASTHLKRKWL EQDESASELR

51 EELNRVNSEN KKLTEMLARV CESYNELHNH LEKLQSRQSP EIEQTDIPIK

101 KRKQDPDEFL GFPIGLSSGK TENSSSNEDH HHHHQQHEQK NQLLSCKRPV

151 TDSFNKAKVS TVYVPTETSD TSLTVKDGFQ WRKYGQKVTR DNPSPRAYFR

201 CSFAPSCPVK KKVQRSAEDP SLLVATYEGT HNHLGPNASE GDATSQGGSS

251 TVTLDLVNGC HRLALEKNER DNTMQEVLIQ QMASSLTKDS KFTAALAAAI

301 SGRLMEQSRT

Zinc finger: $CX_{4-5}CX_{22-23}HXH$

WRKY53 (group III)

1 MEGRDMLSWE QKTLLSELIN GFDAAKKLQA RLREAPSPSS SFSSPATAVA

51 ETNEILVKQI VSSYERSLLL LNWSSSPSVQ LIPTPVTVVP VANPGSVPES

101 PASINGSPRS EEFADGGGSS ESHHRQDYIF NSKKRKMLPK WSEKVRISPE

151 RGLEGPQDDV FSWRKYGQKD ILGAKFPRSY YRCTHRSTQN CWATKQVQRS

201 DGDATVFEVT YRGTHTCSQA ITRTPPLASP EKRQDTRVKP AITQKPKDIL

251 ESLKSNLTVR TDGLDDGKDV FSFPDTPPFY NYGTINGEFG HVESSPIFDV

301 VDWFNPTVEI DTTFPAFLHE SIYY

Zinc finger: $CX_7CX_{23}HXC$

Figure S9: Cys residues in WRKY25, WRKY18 and WRKY53
Amino acid sequences of WRKY25, WRKY18 and WRKY53 are depicted and the DNA-binding domains containing the WRKYGQK and the zinc finger like domain are highlighted in yellow. Additional Cys are highlighted and encircled in red.

Table S1: Oligonucleotides for qRT-PCR

Gene	Atg number	Forward primer 5‘-3‘	Reverse primer 5‘-3‘
WRKY25	Atg30250	GGTTGTGGAGTGAAGAAGCA	TCGTGATTGTGTCTTCCTTCA
WRKY53	At4g23810	ATCCCGGCAGTGTTCCAGAATC	AGAACCTCCTCCATCGGCAAAC
WRKY18	At4g31800	TGGACGGTTCTTCGTTTCTCGAC	TCGTAACTCACTTGCGCTCTCG
WRKY40	At1G80840	AAGCTTCTGACACTACCCTCGTTG	TTGACAGAACAGCTTGGAGCAC
ANAC092	At5g39610	CTTACCATGGAAGGCTAAGATGGG	TTCCAATAACCGGCTTCTGTCG
CAB1	At1g29930	TGCACTACTCAACCTCAATGGC	AAAGCTTGACGGCCTTACCG
SAG12	At5g45890	GCTTTGCCGGTTTCTGTTG	GTTTCCCTTTCTTTATTTGTGTTG
ZAT12	At5g59820	TTGGTTACACGCGCTTTGTTGC	ACAAGCCACTCTCTTCCCACTG
ACTIN2	At3g18780	ACCCGATGGGCAAGTCATCAC	TCCCACAAACGAGGGCTGGA
